# Kinetic and thermodynamic effects of phosphorylation on p53 binding to MDM2

**DOI:** 10.1038/s41598-018-36589-5

**Published:** 2019-01-24

**Authors:** Shilpa Yadahalli, José L. Neira, Christopher M. Johnson, Yaw Sing Tan, Pamela J. E. Rowling, Anasuya Chattopadhyay, Chandra S. Verma, Laura S. Itzhaki

**Affiliations:** 1 Bioinformatics Institute, Agency for Science, Technology and Research (A*STAR), 30 Biopolis Street, Singapore, 138671 Singapore; 2 p53 Laboratory, Agency for Science, Technology and Research (A*STAR), 8A Biomedical Grove, Singapore, 138648 Singapore; 3 Instituto de Biocomputación y Física de Sistemas Complejos, Joint Units IQFR-CSIC-BIFI, and GBsC-CSIC-BIFI, Universidad de Zaragoza, 50009 Zaragoza, Spain; 4 Instituto de Biología Molecular y Celular, Universidad Miguel Hernández, 03202 Elche, Alicante Spain; 5 Medical Research Council Laboratory of Molecular Biology, Francis Crick Avenue, CB2 2QH Cambridge, United Kingdom; 6 Department of Pharmacology, Tennis Court Road, University of Cambridge, CB2 1PD Cambridge, United Kingdom; 7 Department of Biological Sciences, National University of Singapore, 14 Science Drive 4, Singapore, 117543 Singapore; 8 School of Biological Sciences, Nanyang Technological University, 60 Nanyang Drive, Singapore, 637551 Singapore; 9 Present Address: Hutchison/MRC Research Centre, Hills Road, Cambridge, CB2 0XZ United Kingdom

## Abstract

*p53* is frequently mutated in human cancers. Its levels are tightly regulated by the E3 ubiquitin ligase MDM2. The complex between MDM2 and p53 is largely formed by the interaction between the N-terminal domain of MDM2 and the N-terminal transactivation (TA) domain of p53 (residues 15–29). We investigated the kinetic and thermodynamic basis of the MDM2/p53 interaction by using wild-type and mutant variants of the TA domain. We focus on the effects of phosphorylation at positions Thr18 and Ser20 including their substitution with phosphomimetics. Conformational propensities of the isolated peptides were investigated using *in silico* methods and experimentally by circular dichroism and ^1^H-NMR in aqueous solution. Both experimental and computational analyses indicate that the p53 peptides are mainly disordered in aqueous solution, with evidence of nascent helix around the Ser20-Leu25 region. Both phosphorylation and the phosphomimetics at Thr18 result in a decrease in the binding affinity by ten- to twenty-fold when compared to the wild-type. Phosphorylation and phosphomimetics at Ser20 result in a smaller decrease in the affinity. Mutation of Lys24 and Leu25 also disrupts the interaction. Our results may be useful for further development of peptide-based drugs targeting the MDM2/p53 interaction.

## Introduction

The tumor suppressor protein p53 is a transcription factor and a master regulator of cellular function, protecting cells against damage and responding to stress by modulating cell cycle arrest, apoptosis or senescence^1–3^. Hence, it is not surprising that several tumors either carry mutant p53 or are characterized by its functional inactivation^4,5^. This inactivation results from the overexpression of proteins such as E3 ubiquitin ligases that can down-regulate the function of wild-type p53 by its degradation^6^. Indeed, in un-stressed cells, p53 levels are low due to this rapid ubiquitination and degradation.

One such E3 is MDM2 (murine double minute 2). MDM2-deficient lethality in embryonic mice is fully rescued by concomitant deletion of p53^7,8^. Furthermore, MDM2 also promotes its own degradation^9^: under cellular stress, MDM2 is phosphorylated at specific locations and degraded, triggering p53 stabilization^10,11^ and the capability of inducing several genes. MDM2 additionally promotes the export of p53 from the nucleus, thereby reducing its transcriptional activity. In addition, MDM2 inhibits p53 by preventing its interaction with the general transcription machinery, through complex formation. The MDM2/p53 interaction involves at least two regions on both proteins: (i) the DNA-binding domain of p53 binds to the acidic domain of MDM2; and (ii) the TA domain of p53 interacts with the N-terminal domain of MDM2^12,13^. Disruption of these interactions stabilizes p53 and enhances p53 transcriptional activity^14,15^. Therefore, inhibiting the MDM2/p53 interactions is an attractive approach to re-activate p53 in tumors expressing wild-type p53 and where MDM2 gene expression is abnormal, and indeed there are a number of small molecules and constrained peptides in the clinic or in clinical trials for this purpose.

**Figure 1:**
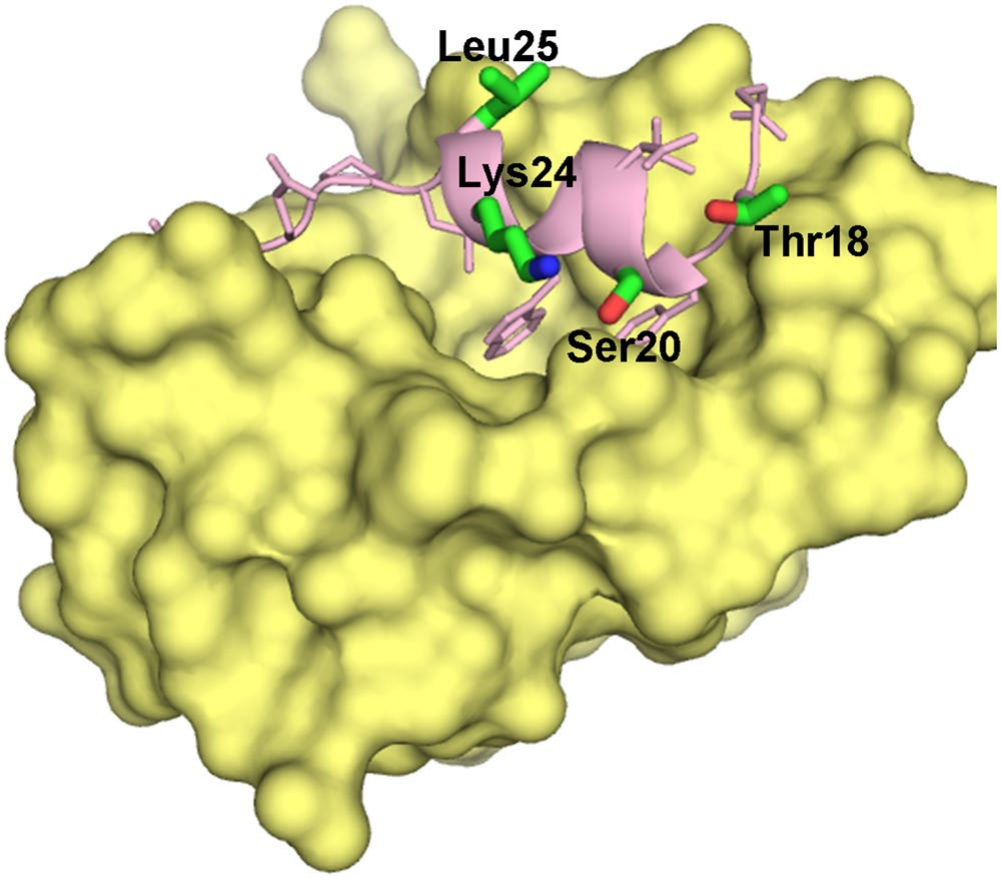
Structure of the p53 peptide (residues 18–29) in complex with the N-terminal domain of MDM2 (PDB ID: 1YCR). MDM2 is shown as a yellow surface and the p53-peptide is shown as light pink cartoon. The side chains of the peptide are shown as sticks. The residues of interest in our study are highlighted and labelled. The residues that are phosphorylated are Thr18 and Ser20. In addition, the effects of mutations at residues Lys24 and Leu25 were also studied. The figure was made using PyMOL (The PyMOL Molecular Graphics System, Version 2.0 Schrödinger, LLC).

The interaction between the N-terminal domain of MDM2 and the TA domain of p53 is well characterised, and there are several nuclear magnetic resonance (NMR) and crystal structures of the MDM2 domain in complex with a variety of p53 peptides and other molecules^16–19^, with the p53 peptide adopting an α-helical conformation in all cases (Fig. 1). These structures show that residues Phe19, Trp23 and Leu26 of the TA, which are part of an amphipathic helix, are inserted into a hydrophobic pocket on the MDM2 surface (Fig. 1). In addition, substitutions at Pro27 and phosphorylation at Thr18 and Ser20 of p53 also modulate the interaction^20–25^.

Here we explored the effects of phosphorylation and of phosphomimetics at positions Thr18 and Ser20 of p53 on the conformational propensities of p53 and the kinetics and thermodynamics of its binding to MDM2. We also investigated how residues Lys24 and Leu25, in combination with the two phosphorylated sites, affected the interaction with MDM2. To this end, we synthesised peptides comprising residues Glu17-Asn29 of the TA region; this region has been shown *in silico* to have a high affinity for MDM2^24^, but this p53 peptide has not been analysed previously by biophysical methods^23^. The p53 peptides were phosphorylated at Thr18 and Ser20 or, alternatively, phosphomimetic substitutions (Thr18Asp, Thr18Glu, Ser20Asp, Ser20Glu) were made at these two positions. Structural studies of the isolated peptides in aqueous solutions were carried out using circular dichroism (CD) and ^1^H-NMR spectroscopy. We also performed stopped-flow fluorescence measurements to determine the kinetic rate constants (*k*_on_ and *k*_off_) of the reaction. Lastly, the equilibrium dissociation constants were measured by isothermal titration calorimetry (ITC) and steady-state fluorescence titrations. To complement the experimental analysis, molecular dynamics (MD) simulations were carried out on the isolated peptides to determine whether there was an intrinsic tendency to populate some residual structure. Our experimental and computational studies show that the isolated peptides have a weak intrinsic tendency to populate β-turn- or α-helix-like conformations around the Ser20-Leu25 stretch. Although we did not observe any changes in the average helical populations in the experiments (CD and NMR), MD simulations of the isolated peptides suggest a shift in the helical populations in the phosphomimetics or phosphorylated species at Thr18. The ITC measurements show that the binding affinity was decreased by 10- to 20-fold when Thr18 was phosphorylated. This decrease in binding affinity was only partially reflected in the dissociation rate constants, which increased by around 2-fold, and there was very little change in the association rates. These results suggest that the binding is not a two-state process, in agreement with our previous computational results^24^. Phosphorylation at Ser20 also resulted in a lower binding affinity, as measured by ITC, but there was no clear correlation between the decrease in binding affinity and changes in *k*_on_ or *k*_off_ values. Control experiments with a peptide containing the phosphomimetics at Thr18 and Ser20 and mutations of Lys24 and Leu25 (to Glu) did not show any binding at all. Therefore, although the binding appears to be modulated substantially by phosphorylation of Thr18, there are other residues within the Glu17-Asn29 region that are also involved.

## Results

### Conformational propensities of the isolated p53 peptides in aqueous solution

**Figure 2:**
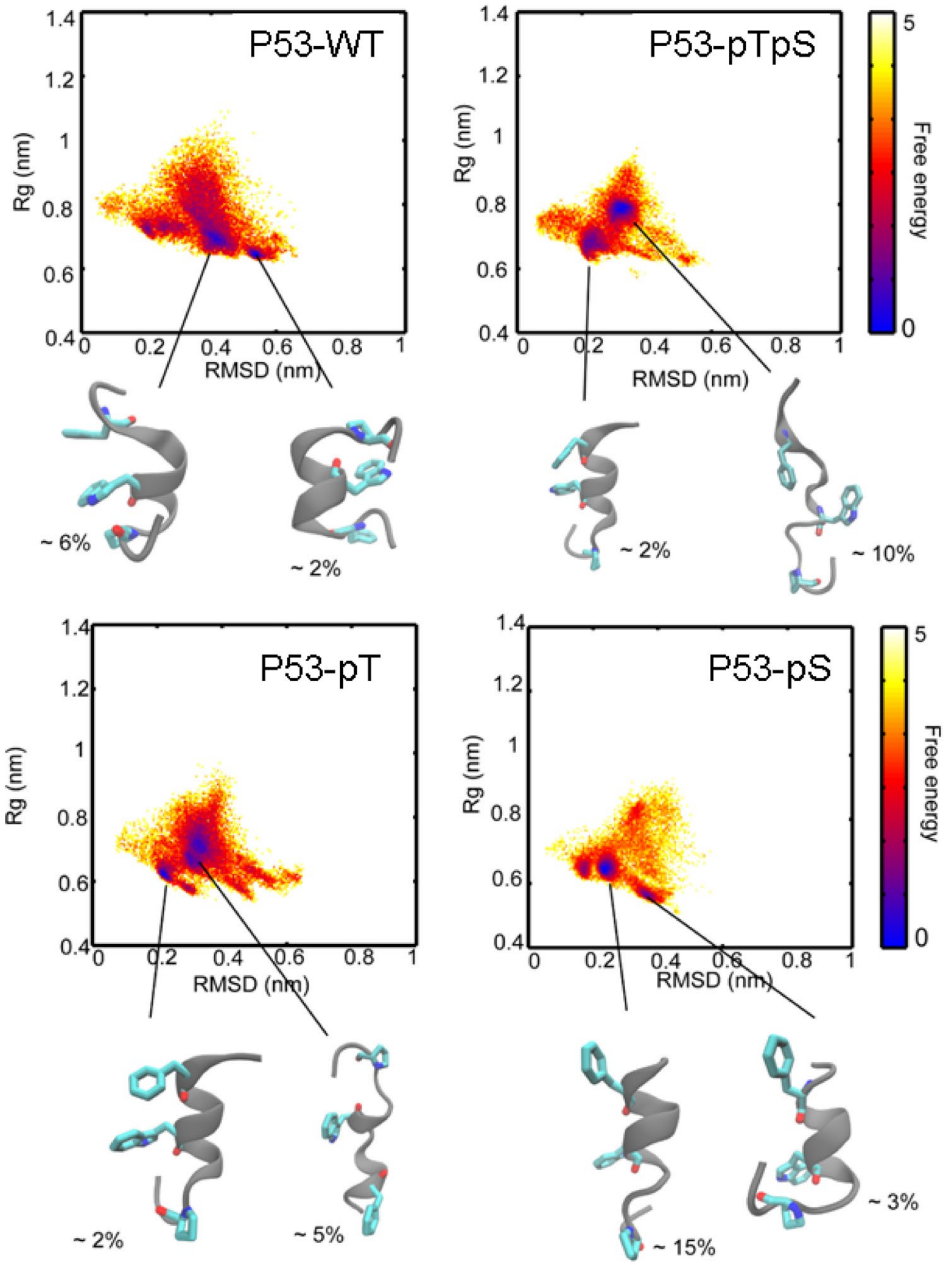
Conformational landscapes of the phosphorylated peptides. 2D FES of WT and phosphorylated p53 peptides sampled during the REMD simulations. The X-axis represents the RMSD of each conformation of a peptide calculated from the bound form of the WT peptide from the crystal structure 1YCR^16^. The Y-axis represents the radius of gyration (*R*_g_) of the peptide. Representative structures from significantly populated clusters are shown in a cartoon representation. Key binding residues Phe19, Trp23, Leu26 are shown as sticks. The percentage of structures in the corresponding clusters are given adjacent to the cartoon. The colors of the free energy surface represent the populations of the peptide conformations, and the color scheme used is shown on the right-hand side; blue and yellow correspond respectively to the highest and lowest density of conformations sampled in our REMD simulations.

As the p53 region under investigation acquires an α-helical conformation upon binding^16^, we wanted to determine the population of folded structures in the peptides, and how this was affected by phosphorylation. The CD spectra of all of the p53 peptides in aqueous solution show an intense band at ~200 nm, with a small shoulder at 222 nm, suggesting that the peptides are mainly disordered (Supplementary Fig. S1A). Only the spectrum of peptide p53-E18-D20-E24-E25 has a maximum band at 222 nm (with a positive value of the ellipticity, Table 1) and a peak with a minimum at 199 nm (Supplementary Fig. S2), that could be due to the presence of polyproline type II structure or β-turn^26^. The spectrum of p53-WT (wild type) is in agreement with that reported in previous work^24^. The molar ellipticity at 222 nm, [Θ]^222^, gives estimates of the percentages of the helical conformations sampled by the peptides^27^ (Table 1) to be less than 12%, and 0% for the p53-E18-D20-E24-E25 peptide. Peptides with the highest helicity (i.e., greater than or equal to 10%, and greater than that of p53-WT) were p53-D18, p53-pS, p53-pT and p53-D18-E20. The deconvolution of the far-UV CD spectra of the peptides, using CDSSTR, CONTIN, Selcon 3 and the k2d programs from the Dichro-Web online server^28,29^, indicated 6–17% of helicity in all the peptides (with 26–40% of random-coil, 15–30% of β-sheet and 20–30% of β-turn). The program k2d indicated low helicity (2–9%) and higher values for random coil (63–90%) compared with the other three programs. Together these results suggest that the helical content in none of the peptides was higher than 17%. In the replica exchange molecular dynamics simulations (REMD), peptides with helicity greater than that of p53-WT were p53-pS, p53-E20 and p53-D20 (Table 1). In addition, the simulations show that phosphorylation of Thr18 (and the phosphomimetics Asp18 and Glu18) results in repulsion with Asp21, thus leading to the loss of the hydrogen bond between the side chains of Thr18 and Asp21, and thereby to a loss of helicity. In contrast, phosphorylation of Ser20, and the concomitant accumulation of negative charge, results in ion pairing with the positively charged side chain of Lys24, which clearly stabilizes the helical state; in fact, in our simulations, p53-pS has the largest population of helical structure. The free energy surfaces (FES) obtained from the REMD simulations of the peptides are shown in Fig. 2. Representative conformations and their percentage populations are also shown. Although there is a general agreement between the simulations and experiments, it is important to note that determining the percentage of helicity of these peptides (either computationally or experimentally) is difficult due to their transient helical nature. In addition, aromatic residues (Phe19 and Trp23 in all the peptides) also absorb at 222 nm^30,31^, which can make it difficult to determine exact helical populations. In conclusion, both the experimental and the computational results indicate that the presence of negative charge at Ser20 (either by phosphorylation or by the phosphomimetic) results in increased helicity of the peptides when compared with p53-WT.

**Table 1:** Helicity and thermodynamic parameters for the p53-peptides from the CD data, TFE-titration curves and MD simulations^a^.

p53-peptide	[TFE]_1/2_ (%)	*m* (cal mol^−1^(%)^−1^)	% Helix in water (from Δ*G* in TFE titrations)^c^	% Helix in water ([Θ]_222_)^d^	% Helix from REMD
Ac-ETFSDLWKLLPEN-NH_2_ (P53-WT)	7 ± 7	130 ± 50	16 ± 3	8.7 (−2741)	7
Ac-E**pt**F**ps**DLWKLLPEN-NH_2_ (P53-pTpS)^b^				8.1 (−2547)	2.1
Ac-E**pt**FSDLWKLLPEN-NH_2_ (P53-pT)^b^				10.2 (−3213)	2
Ac-ETF**ps**DLWKLLPEN-NH_2_ (P53-pS)^b^				14.6 (−4614)	15
Ac-E**e**FSDLWKLLPEN-NH_2_ (P53-E18)	14 ± 5	103 ± 42	7 ± 1	7.5 (−2380)	2.8
Ac-ETF**d**DLWKLLPEN-NH_2_ (P53-D20)	25 ± 3	74 ± 32	4 ± 2	9 (−2811)	7.6
Ac-E**e**F**d**DLWKLLPEN-NH_2_ (P53-E18-D20)	15 ± 1	135 ± 18	3 ± 1	6.6 (−2100)	6.7
Ac-E**e**F**d**DLW**ee**LPEN-NH_2_ (P53-E18-D20-E24-E25)^b^				0 (142.7)	
Ac-E**e**F**e**DLWKLLPEN-NH_2_ (P53-E18-E20)	24.5 ± 0.9	420 ± 390	1	8.7 (−2757)	5
Ac-E**d**FSDLWKLLPEN-NH_2_ (P53-D18)	12.0 ± 0.7	383 ± 142	1	10.5 (−3313)	2.5
Ac-ETF**e**DLWKLLPEN-NH_2_ (P53-E20)	25 ± 2	231 ± 141	1	9.2 (−2911)	10
Ac-E**d**F**e**DLWKLLPEN-NH_2_ (P53-D18-E20)	33 ± 5	112 ± 70	1	11.4 (−3597)	6
Ac-E**d**F**d**DLWKLLPEN-NH_2_ (P53-D18-D20)	9.8 ± 0.8	263 ± 47	1	9 (−2811)	4.7

^a^Experiments were carried out at 5 °C in phosphate buffer (pH 6.8, 50 mM). Data errors are fitting errors to a two-state equation^32^. Mutations (or phosphorylated residues) are indicated in bold in lower case lettering. Final concentrations of the peptides for the titrations were 40 μM.

^b^The TFE-titration curves for the peptides were very flat, i.e. there was a decrease in the ellipticity as the [TFE] increased, but the curves did not have a sigmoidal shape.

^c^Determined from the value of the free energy in aqueous solution (Δ*G* = [TFE]_1/2_ × *m*). For the peptides with a 1% percentage the error was 10%.

^d^Determined from the values of the molar ellipticity at 222 nm (within the parentheses for each peptide), assuming that a 100% helical peptide has a mean residue ellipticity of −31500 deg cm^2^ dmol^−1^ ^27^.

In order to obtain better experimental estimates of the helical content of the p53 peptides, we carried out TFE (2,2,2-trifluoroethanol) titrations^32,33^. Unlike the above programs, which make assumptions about how the ellipticity signal relates to secondary structure content, a TFE titration allows the determination of helical content derived from the free-energy change for the equilibrium between folded (in TFE) and unfolded peptide, without any such assumptions. In addition, the free energy of folding of the peptide can be a useful parameter when interpreting the binding kinetics (see Discussion). The ellipticity at 222 nm increased (in absolute value) for all of the peptides as the TFE concentration was raised (Supplementary Fig. S1B). However, due to the absence of a proper sigmoidal transition for the phosphorylated peptides and the quadruple mutant (Table 1), an estimate of their helical populations could not be obtained. For the rest of the peptides, the midpoint ([TFE]_1/2_, given as a percentage vol:vol) and the *m*-value of the titration curves varied between the different peptides (Table 1). In general, the helical populations of the peptides obtained from TFE titration curves were smaller than those estimated directly from the [Θ]^222^ values, and peptides p53-D20 and p53-E18-D20 showed the greatest helicity. Overall, the CD data (either by direct measurement of [Θ]^222^ or from the TFE titrations) indicate that the p53 peptides are mainly disordered in solution.

**Figure 3:**
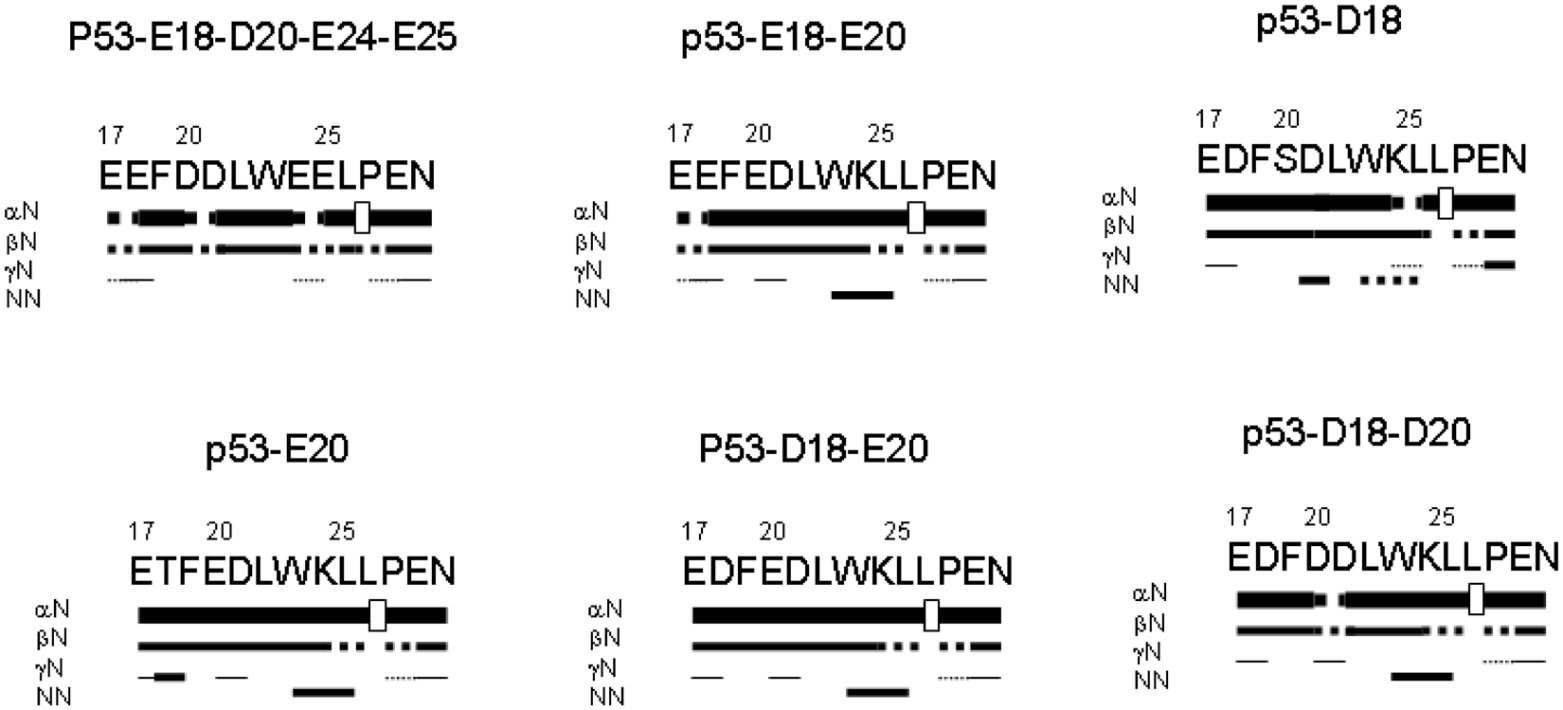
NMR structural characterization of selected p53-peptides: NOEs are classified into strong, medium or weak according to the height of the bar underneath the sequence; signal intensity was judged by visual inspection from the ROESY experiments with 200 ms of mixing time. The corresponding H_α_ NOEs with the following H_δ_ of a proline residue are indicated by an open bar in the row corresponding to the αN(*i*, *i* + 1) contacts. The dotted lines indicate NOE contacts that could not be unambiguously assigned due to signal overlap or diagonal proximity of the chemical shifts. The numbering of residues corresponds to that of the whole sequence of p53.

**Figure 4:**
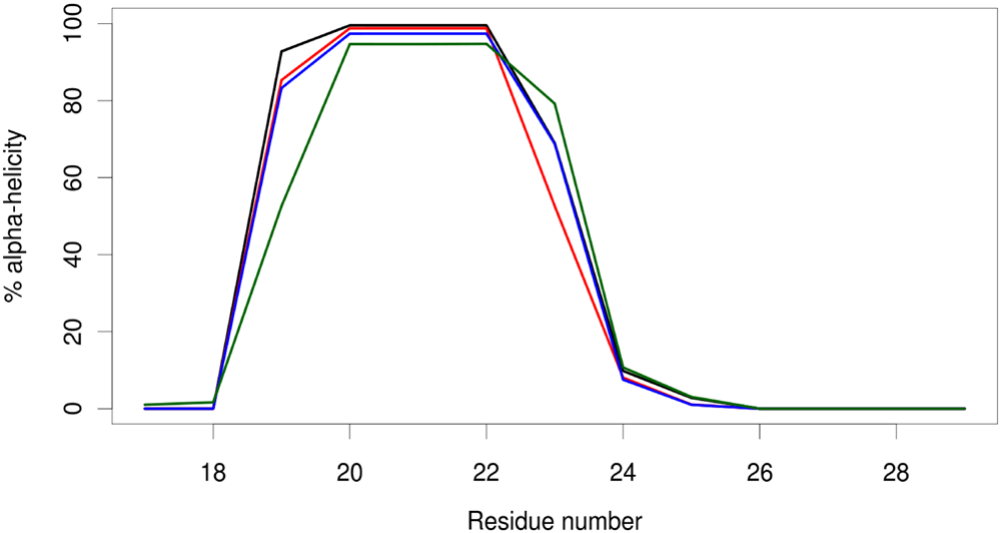
Helix propensities of peptide residues during MD simulations of MDM2-peptide complexes. The peptides are p53-WT (black), p53-pS (red), p53-pT (blue) and p53-pSpT (green).

We also carried out homonuclear 2D ^1^H-NMR experiments to assign all p53 peptides. For all peptides, an NOE (Nuclear Overhuaser effect) between the H_α_ of Leu26 and the H_δ_ of Pro27 was always observed, together with the absence of other minor NOE signals (Fig. 3, Supplementary Fig. S3); these findings suggest that the Leu26-Pro27 peptide bond adopts mainly a *trans* conformation in all the peptides. The peptides are mainly disordered in solution, but there are some residual conformations as suggested by two pieces of evidence. First, the conformational shifts^34^ (Δδ) of H_α_ protons of the region Ser20-Leu25 are outside the commonly accepted range for random-coil peptides (|Δδ| ≤ 0.1 ppm) (Supplementary Tables S1–S13). It is important to note that this region contains Trp23 and is close to Phe19, and therefore, the hydrophobicity of these aromatic residues is an important driving force in the acquisition of local structure in this region (see the results of the MD simulations below). The mean value of those negative conformational shifts was −0.21; by taking into account the average value for fully formed helices (−0.39 ppm)^35^ we estimate that the percentage of secondary structure for *only* those residues is ~50%. However, it must be kept in mind that this value can indicate that half of those residues adopt a fully helical conformation all of the time, or that half of the time all the residues adopt helical conformations. And secondly, sequential NN(*i*, *i* + 1) NOEs (and ROEs) were observed for most of the peptides for residues Asp21-Leu22 and Trp23-Leu25, with the latter not being unambiguously identified in some peptides due to either overlap with other signals or proximity to the spectrum diagonal (Fig. 3 and Supplementary Fig. S3). The only peptide where such NOEs were not observed was p53-E18-D20-E24-E25; interestingly, the conformational shifts for the region Ser20-Leu25 in that peptide were smaller than in the other peptides (Supplementary Tables S1–S13), although the |Δδ| was always lower than 0.1 ppm in that particular region (and therefore, there were some helical conformations around those residues). Thus, as also suggested by the CD data (see above), the quadruple mutant has a lower content of helical populations than the other peptides. Our REMD simulations also indicate that the largest population of helical structure is for the region Phe19-Trp23 (Fig. 4).

There were some differences in the NN(*i*, *i* + 1) NOEs depending on whether Ser20 was phosphorylated or phosphomimicked. The Asp21-Leu22 NOE was not observed for any of the Asp or Glu phosphomimetics at Ser20, suggesting that the accumulation of negative charges is not beneficial for triggering a nascent helix around this residue. In addition, phosphorylation of Ser20 resulted in the appearance of an NOE between pSer20 and Asp21, and the absence of that between Asp21 and Leu22. Taken together, we can conclude that addition of a negative charge at Ser20 yields to shifts in the structured conformational equilibrium of the peptide. On the other hand, addition of a negative charge at Thr18 did not seem to change the presence of sequential NN(*i*, *i* + 1) NOEs, but our REMD simulations suggest that the phosphorylation of Thr18 leads to side-chain repulsion with that of Asp21, resulting in a loss of helicity of those peptides with phosphomimetics (or phosphorylation) at this position (Table 1). Therefore, the presence of that NN(*i*, *i* + 1) NOE in phosphorylated (or phosphomimetics) peptides must be due to a basal, intrinsic conformational preference of the peptides.

The predicted chemical shifts and conformational shifts from the REMD simulation for the ensemble of each peptide were in good agreement with the experimental findings (Supplementary Fig. S4). We used the program SPARTA^36^ to calculate theoretical chemical shifts from the simulations for the H_α_ protons. The values shown are the mean values for each residue from 3000 structures collected from 300 ns of simulation data (Supplementary Fig. S4). These are in good agreement with the experimental chemical shift values. We found some deviations for Phe19 from the experimental values and the maximum difference was seen for the peptides p53-pT and p53-pTpS. This could be due to ring-current effects^34^. We also saw some differences in the hydrogen-bonding pattern and the backbone torsional distributions when the residues are phosphorylated (Supplementary Fig. S5). Interestingly, when Thr18 is phosphorylated (either alone or together with Ser20), the hydrogen bond between Thr18 and Asp21 side-chains that is seen in p53-WT is broken, and yet the two residues still interact but through sodium ions or water molecules (Supplementary Fig. S6).

### Kinetics and thermodynamics of the MDM2/p53 interaction

To characterize the binding kinetics and thermodynamics of the wild-type and mutant p53 peptides with MDM2, we used three orthogonal techniques. First, we determined the kinetic rate constants (*k*_on_ and *k*_off_) using stopped-flow fluorescence (similar to the method employed previously by Fersht and co-workers^23^). Second, for selected peptides we determined the dissociation constant, *K*_d_, using fluorescence titrations. And third, we used ITC to determine the thermodynamic parameters for binding. For both kinetic and equilibrium fluorescence measurements, we used the intrinsic fluorescence of Trp23 in the p53 peptide, which has been shown previously to be a good probe of binding to MDM2 (as MDM2 has no tryptophan residues)^23^.

**Figure 5:**
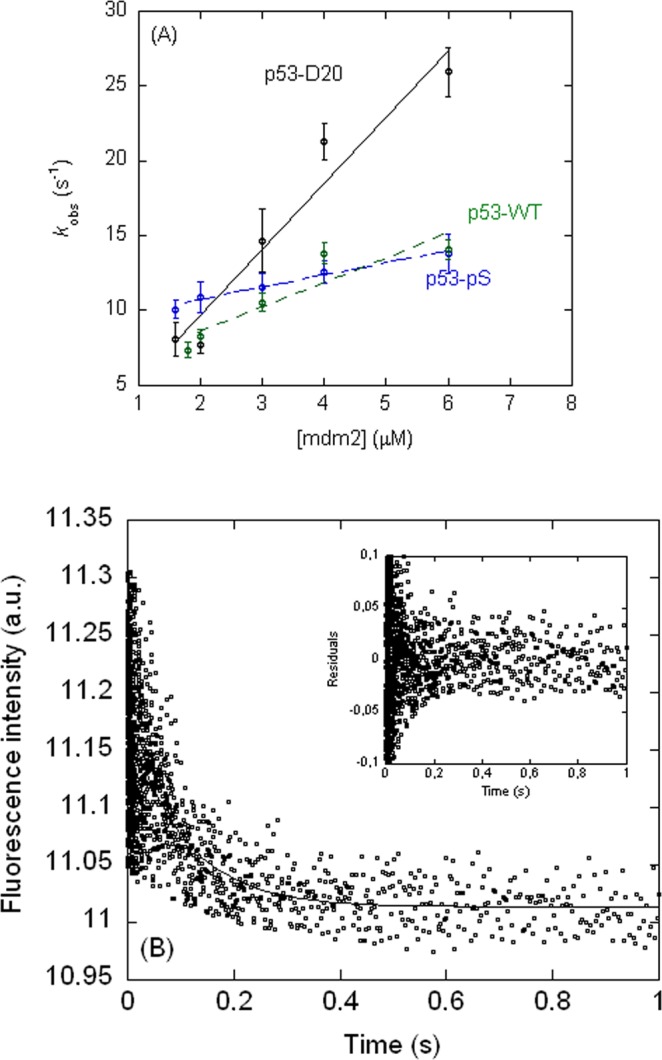
Stopped-flow fluorescence measurements of the binding kinetics of selected p53 peptides to MDM2: (**A**) Pseudo-first order plots of the observed rate constants as a function of increasing MDM2 concentration, with the p53-peptide concentration kept constant. Errors for each *k*_obs_ (s^−1^) are those obtained from fitting of the kinetic traces to a single exponential function. (**B**) Kinetic trace for binding of p53-WT to MDM2 at a concentration of 4 μM (the residuals are shown in the inset). Experiments were carried out at 15 °C.

The kinetic parameters (Table 2, Fig. 5) show that the association rate constant (*k*_on_): (i) increased when Thr18 was mutated to either Asp or Glu; (ii) increased when Ser20 was mutated to either Asp or Glu, and the changes were larger than those in Thr18 mutations; and, (iii) increased in the double phosphomimetics, but the changes depended on whether there was Asp or Glu at position 18. On the other hand, the dissociation rate constant (*k*_off_): (i) increased for all the single phosphomimetics or phosphorylated species at Thr18, and the variations were larger than for *k*_on_; and (ii) remained the same or decreased slightly for all of the single phosphomimetics and the phosphorylated variant at Ser20. *K*_d_ values can be calculated from the ratios of the kinetic rate constants, although these values have large errors due to the long extrapolation required for *k*_off_ (the y-axis intercept). The smallest *K*_d_ values were those of the single phosphomimetics at Ser20 (Table 1). The quadruple mutant did not show any evidence of binding under our conditions; therefore, as there was binding observed for the double phosphomimetic mutant, p53-E18-D20, our results indicate that Lys24 and Leu25 are also important for binding even though they are not directly involved in the interaction with MDM2^16^.

**Table 2:** Kinetic and equilibrium (fluorescence) data for the p53-peptides and MDM2 binding reaction^a^.

p53-peptide	*k*_on_ 10^6^ (M^−1^ s^−1^)	*k*_off_ (s^−1^)	*K*_d_ (k_off_/k_on_) (μM)^c^	*K*_d_ (μM)^d^
Ac-ETFSDLWKLLPEN-NH_2_ (P53-WT)^c^	1.6 ± 0.1	9.0 ± 0.3	5.5 ± 0.5	3.0 ± 1.2
Ac-E**e**FSDLWKLLPEN-NH_2_ (P53-E18)	3.8 ± 0.7	16 ± 2	4.2 ± 0.9	2.3 ± 0.6
Ac-ETF**d**DLWKLLPEN-NH_2_ (P53-D20)	9.5 ± 0.9	7 ± 2	0.7 ± 0.2	1.5 ± 0.4
Ac-E**e**F**d**DLWKLLPEN-NH_2_ (P53-E18-D20)	4 ± 2	18 ± 6	4.0 ± 3.0	8.3 ± 4.9
Ac-E**e**F**d**DLW**ee**LPEN-NH_2_ (P53-E18-D20-E24-E25)^b^				
Ac-E**e**F**e**DLWKLLPEN-NH_2_ (P53-E18-E20)	2.7 ± 0.9	17 ± 2	6 ± 2	
Ac-E**d**FSDLWKLLPEN-NH_2_ (P53-D18)	5 ± 3	23 ± 9	5.0 ± 3.0	
Ac-ETF**e**DLWKLLPEN-NH_2_ (P53-E20)	9 ± 2	8 ± 5	0.9 ± 0.6	0.7 ± 0.1
Ac-E**d**F**e**DLWKLLPEN-NH_2_ (P53-D18-E20)	8 ± 2	13 ± 4	1.6 ± 0.6	3.2 ± 0.9
Ac-E**d**F**d**DLWKLLPEN-NH_2_ (P53-D18-D20)	11 ± 4	20 ± 11	1.8 ± 1.2	
Ac-ETF**ps**DLWKLLPEN-NH_2_ (P53-pS)	3.3 ± 0.8	5 ± 1	1.6 ± 0.6	0.3 ± 0.1
Ac-E**pt**FSDLWKLLPEN-NH_2_ (P53-pT)	2.8 ± 1.6	20 ± 3	7.0 ± 4.0	3.0 ± 1.6
Ac-E**pt**F**ps**DLWKLLPEN-NH_2_ (P53-pTpS)	1.9 ± 1.1	11 ± 2	5.7 ± 3.5	2.5 ± 0.9

^a^Experiments were carried out at 15 °C. Data errors are fitting errors from the slope and the y-axis intercept of the pseudo-first-order plots. Mutations are indicated in bold, in lower case lettering. Final concentrations of the peptides into the stopped-flow chamber were 0.125 μM.

^b^No exponential behaviour was observed in any trace at any of the concentrations of MDM2 tested.

^c^Determined assuming a two-state binding reaction without any intermediates.

^d^Determined from fluorescence titrations. The change in fluorescence at 315 nm (after excitation at 280 nm) as a function of p53-peptide concentration was fitted to Eq. (1).

Because of the large errors that result when determining the *K*_d_ values from ratio of the kinetic rate constants, we instead looked at determining these values for a subset of peptides directly from equilibrium titrations monitoring intrinsic p53-peptide fluorescence. The errors in the *K*_d_ values obtained were large due to the small amplitude of the changes (Supplementary Fig. S7). Nevertheless, the results (Table 2, Supplementary Figs S7 and S8) indicate that the *K*_d_ values for the mutants were generally slightly larger than, but still within the error of, that for p53-WT.

**Figure 6:**
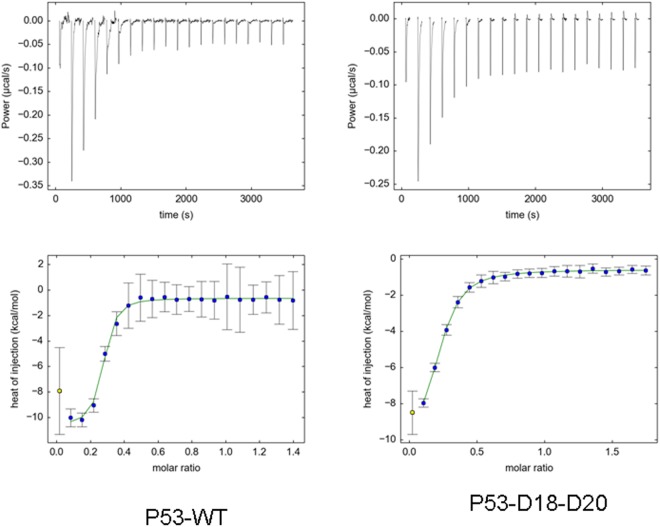
ITC of p53-peptides and MDM2: The upper traces show the original raw data, and the lower traces represent the fit after integration carried out by NITPIC software. The integrated peaks are shown as: (i) yellow squares, for the first point peak; and (ii) blue circles, for the rest of the integrated peaks. Traces were obtained with MDM2 in the reaction cell and the p53 peptide titrated into the cell. Experiments were carried out at 15 °C.

We were able to obtain much more accurate *K*_d_ values by using ITC. The results are given in Table 3, and representative data are shown in Fig. 6. Since ITC is the gold-standard^37^ for determining binding affinities, we refer to these *K*_d_ values in our subsequent discussion. The *K*_d_ for p53-WT is similar to that measured previously for the same peptide (Glu17-Asn29) under similar solution conditions: 1.54 ± 0.09 μM^24^, but larger than for other p53 peptides of different lengths: 0.5 μM (Ser15-Asn29) and 0.046 μM (Glu17-Leu26)^23^. The ITC results show that the *K*_d_ values of all the mutants increased relative to the p53-WT; these changes were largest for the phosphomimetics and the phosphorylated variants at Thr18. This result highlights the importance of this residue for the binding and is in agreement with a previous study which showed that only the phosphorylation of Thr18 is responsible for abrogating p53–MDM2 binding^23^. There were differences between the values obtained by ITC (Table 3) and fluorescence (Table 2). However, they can be rationalised as follows: steady-state techniques, in which the observable is the equilibrium state after long incubation times allowing optimal accommodation of the interacting molecules (e.g. fluorescence titration), may give rise to higher affinities than transient event techniques, where the observable mainly reflects the first encounter between the interacting molecules (e.g. ITC) and therefore slow conformational rearrangements may be overlooked. Similar differences in the measured binding affinities between different techniques have been observed when measuring interactions in other proteins and peptides^38–40^. Interestingly, the binding of p53-pTpS to MDM2 is associated with a positive ∆*S* value (as measured by ITC, Table 3), which indicates that there is an increase in entropy upon complex formation, whereas the formation of the other MDM2-peptide complexes is associated with negative ∆*S* values. MD simulations of the MDM2 complexes with wild-type p53 and phosphorylated p53 peptides suggest that the differences in the ∆*S* values could be attributed to the different helix propensities of the bound peptide. The higher the α-helicity of the peptide in the bound state, the greater the entropy loss upon binding. Wild-type p53 has the highest overall helix propensity in the complex simulations, followed by p53-pS, p53-pT and finally p53-pTpS (Fig. 4). This suggests that wild-type p53 incurs the greatest entropy loss, followed by p53-pS, p53-pT and p53-pTpS, and this trend is reflected in the ∆*S* values obtained from ITC (Table 3). The simulations show that electrostatic repulsion between the negatively charged pT18 and pS20 perturbs the helical structure of bound p53-pTpS, thus accounting for its low helix propensity.

**Table 3:** Calorimetric data for the p53-peptide and MDM2 binding reaction^a^.

p53-peptide^a^	Δ*H* (kcal mol^−1^)^b^	Log *K*_A_^b^ (*K*_d_ = 1/*K*_A_ (μM))	*T*Δ*S* (kcal mol^−1^)^b^
Ac-ETFSDLWKLLPEN-NH_2_ (P53-WT)	−10.07	5.73 (1.8)	−3.23
Ac-E**e**FSDLWKLLPEN-NH_2_ (P53-E18)	−7.02	5.15 (7.1)	−0.26
Ac-ETF**d**DLWKLLPEN-NH_2_ (P53-D20)	−10.24	5.24 (5.7)	−3.36
Ac-E**e**F**d**DLWKLLPEN-NH_2_ (P53-E18-D20)	−8.80	4.69 (20.4)	−2.64
Ac-E**e**F**d**DLW**ee**LPEN-NH_2_ (P53-E18-D20-E24-E25)	−^c^	−^c^	−^c^
Ac-E**e**F**e**DLWKLLPEN-NH_2_ (P53-E18-E20)	−9.12	5.30 (5.0)	−2.16
Ac-E**d**FSDLWKLLPEN-NH_2_ (P53-D18)	−18.39	4.72 (19.0)	−12.19
Ac-ETF**e**DLWKLLPEN-NH_2_ (P53-E20)	−^c^	−^c^	−^c^
Ac-E**d**F**e**DLWKLLPEN-NH_2_ (P53-D18-E20)	−9.90	5.23 (5.9)	−3.03
Ac-E**d**F**d**DLWKLLPEN-NH_2_ (P53-D18-D20)	−9.95	4.56 (27.5)	−3.96
Ac-ETF**ps**DLWKLLPEN-NH_2_ (P53-pS)	−9.73	5.71 (1.9)	−2.26
Ac-E**pt**FSDLWKLLPEN-NH_2_ (P53-pT)	−6.83	4.60 (25.1)	−0.79
Ac-E**pt**F**ps**DLWKLLPEN-NH_2_ (P53-pTpS)	−4.81	4.60 (25.1)	1.23

^a^Mutations are indicated in bold, in lower case lettering.

^b^The free energy values from ITC are given for the binding reaction, except for the *K*_d_ values, which are listed in parentheses in the second column next to the *K*_A_ values.

^c^Not determined.

## Discussion

### Structural properties of the isolated p53 peptides

In previous studies of isolated peptide fragments of the TA domain of p53 in aqueous solution, it has been shown that this region is mainly unfolded, although there is evidence of structured conformations around residues Phe16 and Trp23^25,41–43^. In addition, these residues are important in binding to MDM2^16,17^. Here we have observed that the region Ser20-Leu25, in the peptide Glu17-Asn29, populates a β-turn or α-helix-like conformation, as suggested by: (i) the NMR conformational shifts (Supplementary Tables S1–S13, and Supplementary Fig. S4); (ii) the presence of sequential NN(*i*, *i* + 1) NOEs (Fig. 3); and (iii) the helical populations estimated from REMD (Fig. 4). However, it is important to point out that this population is very small, as evidenced by: (i) the percentages of helical structure determined by CD (always less than 10%, Table 1); and (ii) the absence of medium-range contacts (i.e., αβ(*i*, *i* + 3) NOEs) in that polypeptide region.

The unambiguous presence of folded conformations, despite their low population, was also benchmarked by our REMD simulations. There were subtle differences between the *in silico* results and those from the NMR and CD experiments; for instance, although we observed in our REMD simulations that the presence of a negative charge at Thr18 decreased the helical population (Table 1) due to the absence of a hydrogen bond involving the side chain of Thr18, the CD and the NMR data (NOEs and conformational shifts) did not indicate any change in the population of helical structure. It is likely that by CD we did not observe any variation because we can only detect the average of all populations in solution, where changes in population are masked by the large population of the dominant conformation (or even by the bands of aromatic residues). However, it is not clear how to rationalize the differences in the population of helical structures observed by the NMR and the REMD (Table 1), especially since our REMD simulations predict quite well the conformational shifts of the backbone H_α_ (Supplementary Fig. S4). We hypothesize that the presence of the aromatic residues (even taking into account their effect on the calculation of conformational shifts) could alter the estimated helical populations by NMR; furthermore, the presence of such hydrophobic aromatic residues, which are key to induce binding^16^, are also important in shifting the population of folded structures (β-turn, α-helix-like, or polyproline type II). Alternatively, we also suggest that as the increase (or decrease) of helical populations is favoured by hydrogen bond formation among several side chains (see above), the variation of the values of the chemical shifts of the backbone protons compared to p53-WT in the more helical conformations could be small.

### Molecular basis of MDM2/p53 complex formation: kinetics and thermodynamics

Thr18 is a highly conserved residue among eukaryotic p53 proteins^23^. We observed that the phosphorylation of Thr18 has a large detrimental effect on binding affinity of the p53 peptide for MDM2 (Tables 2 and 3), although the low micromolar values observed are still in the range of those observed for other protein-protein interactions (PPIs)^44^. This decrease in affinity was only partially reflected in the dissociation rate constant, which increased by around 2-fold, and there was very little change in the association rate constant. Likewise, the *k*_off_ values of the phosphomimetic variants at Thr18 also increased relative to p53-WT, and there were only small changes in the *k*_on_ values (Table 2). Phosphorylation also appeared to shift the population of folded conformations in the peptide as shown by REMD simulations (Table 1). The p53-pT peptide was significantly less helical than the p53-WT; and p53-pS was more helical than the p53-WT in MD simulations of the isolated peptides. These results agree with those published previously with shorter peptides^23^, and they explain the lower measured affinity of phosphorylated species of full-length p53 for MDM2^22,45^.

The phosphomimetics and phosphorylated variant at Ser20 behaved differently from those at Thr18. The phosphorylated variant had a similar binding affinity to that of p53-WT (Table 3), and there were only small changes in *k*_on_, and *k*_off_ decreased (Table 2). The phosphomimetics showed significant increases (up to 6-fold) in the *k*_on_ values, and only small changes in *k*_off_ (Table 2). The major contribution to the behaviour of the double phosphomimetics came from Thr18. Therefore, it appears that at Ser20, the long alkyl groups in the side chains of the phosphomimetics impose a steric hindrance on the interaction, making them not ideal at mimicking the effects of phosphorylation (see also above).

Our studies also show that mutation of Lys24 and Leu25 (to Glu residues), together with those at Thr18 and Ser20, disrupted binding (Table 2). Thus, it is not only Phe19, Trp23 and Leu26 that are important for binding to MDM2, but other residues located at the C-terminus of that patch also contribute. It has been shown both *in silico*^24,46^ and experimentally^25,47^ that binding to MDM2 is also modulated by C-terminal residues. This effect may arise because residues Lys24 and Leu25 strengthen the helical region formed upon binding of p53 by favouring the helical amphipathy and the side-chain hydrogen bonding (see Results section). We know from our REMD simulations that phosphorylation of Ser20: (i) results in salt-bridge formation with the side-chain of Lys24; and, (ii) is associated with increased helicity (as seen for the phosphomimetics at this position).

As the isolated p53 peptides have very little helical structure whereas they have a folded helical conformation in the crystal structures of the p53-MDM2 complex^16^, the measured binding thermodynamics and kinetics must take into account the effects of peptide folding as well as binding. Two mechanisms are possible. According to a “conformational selection” mechanism, there are two consecutive equilibria in which the helical form of the peptide is the only one capable of binding to MDM2:

$$p53-\mathop{peptide}\limits_{(unfolded)}+MDM2\underset{\mathop{\longleftarrow }\limits_{{k}_{u}}}{\overset{{k}_{f}}{\longrightarrow }}p53-\mathop{peptide}\limits_{(helical)}+MDM2\underset{\mathop{\longleftarrow }\limits_{{k}_{off}}}{\overset{{k}_{on}[p53-peptid{e}_{helical}]}{\longrightarrow }}MDM2:\mathop{p53}\limits_{(helical)}-peptide$$

According to an “induced-fit” mechanism^48^, the binding involves the following pathway:

$$\mathop{MDM2}\limits_{(free)}+p53-peptide\underset{\mathop{\longleftarrow }\limits_{{k}_{off}}}{\overset{{k}_{on}[p53-peptide]}{\longrightarrow }}MDM2:p53-peptid{e}^{\ast }\underset{\mathop{\longleftarrow }\limits_{{k}_{off}^{\ast }}}{\overset{{k}_{on}^{\ast }}{\longrightarrow }}MDM2:\mathop{p53}\limits_{(complex)}-peptide$$

where the species with an asterisk is an intermediate formed after binding to MDM2 with a different population of helical structure from that of the isolated p53 peptide in solution. We have observed in recent MD simulations that the bound peptide acquires varied helical turns throughout its length, consistent with an “induced fit” mechanism of binding^24,49^. Alternatively, a “conformational selection” mechanism^48^ might be favored because of the intrinsic propensity of the p53 peptide to adopt a folded conformation around Trp23, and this hypothesis has been tested in our recent MD studies with p53 peptides of different length^24^. However, these *in silico* results suggest that even in the short peptides used in the present study, the reaction follows neither a simple “conformational selection” nor an “induced-fit” mechanism. We can also comment on these questions in light of our experimental results, although a more detailed kinetic study beyond the scope of the present work would be required to attempt to unambiguously assign a mechanism. The *K*_d_, obtained from the ratio of the kinetic rate constants, *k*_on_ and *k*_off_, (5.5 μM for p53-WT, Table 2) is very different from that obtained directly by equilibrium measurement (by ITC) (1.8 μM for WT peptide, Table 3). This result suggests that the mechanism of association is not a simple one-step process of assembly, and it suggests that there could be process before and/or after the major encounter, likely to be related to the malleability of the p53 peptide and its interface with MDM2 as highlighted by the extensive computational analysis of this fascinating (and biologically pivotal) and complex multi-domain protein-protein interaction system^50–52^.

### Are glutamate and aspartate good mimics of phosphorylated serine and threonine residues?

Although the phosphomimetics used at Thr18 and Ser20 broadly reproduced the changes in the affinity between p53 peptides and MDM2 (Table 3), there were subtle variations between the Ser20 phosphomimetics and the phosphorylated variant: (i) the NOE pattern (Fig. 3 and Supplementary Fig. S3) changed, with the appearance of a new NN(*i*, *i* + 1) NOE; (ii) the *k*_on_ values were larger for the phosphomimetics (Table 2) than for the phosphorylated variants; and (iii) for the phosphorylated variants, the *K*_d_ values were similar to that of p53-WT, whereas they were larger for the phosphomimetics (Table 3). The first observation implies that the population of folded conformations in the phosphorylated peptides was skewed by the presence of phosphate. This change in folded conformations was observed in our REMD simulations (see above and Supplementary Fig. S5) but not in the NMR or CD results. And the second difference could be explained as due to the bulkier size of the phosphate moiety in a smaller region than the environment around the side-chains of Glu and Asp residues; that is, there was a steric hindrance of pSer when the peptide was close to MDM2 (a similar steric hindrance was observed in the isolated double phosphorylated peptides during our REMD simulations). Therefore, taken together, our results suggest that the use of phosphomimetics is not ideal for studies of phosphorylated peptides.

### Perspectives on the design of helical peptides for inhibition of PPIs

Although in general the prospects for the therapeutic application of peptides is limited due to poor cell penetration and high propensity for proteolysis and metabolic degradation, there are several FDA-approved peptides for therapeutic use^53^ with others in the process of approval including a p53-based peptide^54^. Such peptides are usually constrained into the bioactive conformation using chemical crosslinking, which also makes them more resistant to degradation and they are better suited as inhibitors of protein-protein interactions (PPIs)^44^. In those examples, peptides are designed based on similarity with the sequence of the partner protein. Our experimental and *in silico* studies suggest that helicity is not the sole factor governing the MDM2-binding affinity of p53 peptides (Table 1) but rather, there are other additional factors that come into play, such as peptide amphipathicity, the potential for hydrogen-bond formation and even the presence of residual plasticity in p53^47^, all of which are optimal in the wild-type p53 sequence. Finally, it is important to note that the role of phosphorylation in biology is ubiquitous and key to turning on and off several signalling pathways, and generally, the molecular effect of phosphorylation is to modulate a protein-protein interaction as is the case here. A deeper understanding of the structure-activity relationships of peptides with phosphorylation sites may enable the engineering of features into peptides to modulate signals either as a probe or as a therapeutic. This latter assumes significance in light of a resurgence in using peptides as therapeutics.

## Materials and Methods

### Materials

Deuterium oxide was obtained from Apollo Scientific (UK). All Fmoc-protected amino acids, hydroxybenzotriazole (HOBt), O-Benzotriazole-N,N,N′,N′-tetramethyl-uronium-hexafluoro-phosphate (HBTU) and Rink amide resin were purchased from Novabiochem (Germany). Trifluoroacetic acid (TFA), anhydrous ethyl ether, sodium trimethylsilyl [2,2,3,3–^2^H_4_] propionate (TSP), deuterated acetic acid and its sodium salt were purchased from Sigma Aldrich (USA). Dichloromethane (DCM), diisopropylethylamine (DIPEA), *N*,*N*-Dimethylformamide (DMF), piperidine and HPLC grade acetonitrile were obtained from Merck (Germany). TCEP (Tris(2-carboxyethyl)phosphine) was from VWR (UK). Thrombin was from GE Healthcare (UK). Standard suppliers were used for all other chemicals. Water was deionized and purified on a Millipore system.

### Peptide synthesis

Acetylated and amidated peptides were synthesized and purified as described^24^. It has been observed that shorter p53-peptides, containing the interacting residues, have a higher affinity than longer ones^23^. The peptides comprised residues Glu17-Asn29, since this polypeptide contains the key residues for binding to MDM2 (Phe19, Trp23 and Leu26) and the two phosphorylatable residues: Thr18 and Ser20.

The p53 peptide with the wild-type sequence, the phosphorylated peptides at Thr18 and Ser20, and the double phosphorylated one at Thr18 and Ser20 were purchased from Genscript (New Jersey, USA) with a purity higher than 95% as determined by mass spectrometry; phosphorylation at those positions was confirmed by mass spectrometry and NMR spectroscopy (see below for further details). In all cases, purity of peptides was also checked by SDS gels. The sequences of the peptides are indicated in Table 1. Peptide concentrations were determined from the absorbance of Trp23^55^.

The rationale behind the mutations selected was the following. We were interested in finding out how phosphorylation at either Thr18 or Ser20 positions, or at both, affected the kinetics and thermodynamics of the binding to MDM2. We were also interested in how phosphomimetics (i.e., mutations at Asp or Glu of phosphorylation sites) could mimic the real phosphorylation, and therefore we synthesised: (i) the two variants at both positions; and (ii) the four possible double mutants at those same sites. Lastly, based on our previous MD simulations^24^, we were interested in determining how positions Lys24 and Leu25 affected the binding together with the phosphomimetics at positions Thr18 and Ser20.

### Expression and purification of MDM2

Expression and purification of human MDM2 (residues 2–125) was carried out as described^23^ in BL21 (DE3) cells. Protein purity was higher than 95% as determined by SDS PAGE gels. Protein concentration was determined from the absorbance of its seven Tyr residues^55^.

### Fluorescence spectroscopy

The MDM2 construct has only tyrosine residues and no tryptophan residues, whereas the p53 peptides contain a single tryptophan, the fluorescence of which has been shown previously to be capable of reporting on the binding (both at equilibrium and under kinetic conditions)^23^. Fluorescence spectra were collected on a Perkin Elmer LSB55 spectrofluorimeter (Perkin Elmer, USA), interfaced with a bath, at 15 °C. Experiments were carried out at pH 8.0 (50 mM Tris-HCl buffer), 250 mM NaCl, 0.5 mM TCEP. The samples were prepared the day before and left overnight at 5 °C to reach equilibrium and then incubated for 1 h at 15 °C. The samples were left overnight to allow the system to equilibrate following standard procedures in our laboratories. The titrations were carried out at 15 °C rather than 25 °C due to the tendency of MDM2 to aggregate at temperatures above 15 °C.

For the fluorescence titrations of selected p53-peptides and MDM2 to determine the dissociation constant, increasing amounts of the corresponding peptide, in the range 0–10 μM, were added to a solution with a fixed concentration of MDM2 (4 μM). Excitation wavelengths were 280 and 295 nm with excitation and emission slits of 5 nm. The fluorescence intensity values of a blank solution containing peptide only were subtracted from each point. The dissociation constant of the MDM2/p53peptide complex, *K*_d_, was calculated by fitting the changes in the fluorescence intensity at a particular wavelength *versus* the concentration of added p53 peptide to the following equation^56,57^:

1 $$F={F}_{0}+\frac{{\rm{\Delta }}{F}_{\max }}{2{[MDM2]}_{T}}[({[MDM2]}_{T}+{[{\rm{p53}}-{\rm{peptide}}]}_{T}+{K}_{d})-{(\begin{array}{c}{({[MDM2]}_{T}+{[{\rm{p53}}-{\rm{peptide}}]}_{T}+{K}_{d})}^{2}\\ -4{[MDM2]}_{T}{[{\rm{p53}}-{\rm{peptide}}]}_{T}\end{array})}^{1/2}]$$

where *F* is the measured fluorescence intensity at any particular concentration of p53 peptide after subtraction of the blank; Δ*F*_max_ is the maximal change in the fluorescence intensity of the p53 peptide when all of MDM2 is forming the complex compared to the fluorescence of isolated p53 peptide; *F*_0_ is the fluorescence intensity when no p53 peptide was added; [MDM2]_T_ is the total concentration of MDM2 (4 μM); and [p53-peptide]_T_ is that of p53 peptide, which is varied during the titration. For each peptide, the titration was repeated twice. At all concentrations, the absorbance of p53 peptide was kept lower than 0.2 units of absorbance (at 280 nm) to avoid inner-filter effects during fluorescence excitation^58^.

### CD spectroscopy

Spectra were acquired on a Jasco J815 spectropolarimeter (Jasco, Japan) interfaced with a Peltier unit. The instrument was periodically calibrated with (+) 10-camphorsulphonic acid. Far-UV measurements (in aqueous or TFE solutions) were performed in 0.1-cm-pathlength quartz cells (Hellma), with a response time of 2 s, a band width of 1 nm, data were collected every 0.2 nm, and a scan velocity of 50 nm/min (or 20 nm/min). Six (or five) scans were averaged and collected between 195 and 250 nm or between 190 nm and 260 nm (for the highest concentration assayed). In all peptides, two concentrations (10 μM and 70 μM) in aqueous solutions (50 mM sodium phosphate buffer, pH 6.8 2,5 °C) were used to determine whether either the shapes or the intensities of the spectra were concentration-dependent. We did not observe any difference in the shape or intensity of the spectra for any of the thirteen peptides at the studied concentrations. In all cases (aqueous solution or TFE titrations), the corresponding blank solutions (containing only buffer, or buffer and the corresponding amount of TFE) were subtracted from the spectra.

For the TFE titrations, a peptide concentration of 40 μM was used. The samples were prepared the day before and left overnight at 5 °C to reach equilibrium. The experiments in aqueous solution were acquired at 5 °C, in 50 mM sodium phosphate buffer, pH 6.8. Raw ellipticity was converted to molar ellipiticity, [Θ], from which the percentage of helical populations was determined^27^. Co-solvent concentrations were indicated in percentage of volume. The helical population for each peptide in aqueous solution was determined assuming a two-state equilibrium for the helix ↔ random-coil transition induced by TFE, as suggested by the presence of an isodichroic point^32,33^. Fitting of the titration curves, obtained by observation of the molar ellipticity at 222 nm as the TFE concentration was increased, was carried out with Kaleidagraph (Synergy software).

### NMR spectroscopy

NMR experiments were performed at 10 °C on a Bruker Avance DRX-500 spectrometer (Bruker GmbH, Germany), equipped with a triple resonance probe and z-pulse field gradients. Temperature of the probe was calibrated with methanol^59^. All experiments were carried out at pH 4.5 (50 mM acetate buffer), by adding the corresponding amount (50 μL) of a stock solution of 0.5 M acetic deuterated buffer in D_2_O. We carried out the experiments at that pH to allow for the detection of the largest possible number of amide protons. The pH of the samples was measured before and after the experiments with an ultra-thin electrode (Sigma-Aldrich). Resonances were referenced to external TSP taking into account the pH-dependence of its signal^59^. The final concentration for any of the peptides was in the range 1.5–2 mM.

- 1*D-*^1^*H-NMR spectra*- An amount of 256 scans were acquired with 32 K acquisition points for the homonuclear 1D-^1^H-NMR spectra. Spectra were processed with TopSpin 2.1 (Bruker GmbH, Germany), after zero-filling. The line-width of the spectra of any peptides was that expected from their molecular weights, and then, there was no evidence of any self-association equilibrium for any of the peptides, further supported by the CD findings.
- *2D-*^*1*^*H-NMR spectra-* Two-dimensional experiments with a spectral width of 7801.69 Hz in each dimension were acquired in the phase-sensitive mode by using the time-proportional-phase incrementation technique (TPPI)^60^. Standard DQF-COSY (double quantum-filter correlation spectroscopy), TOCSY (80 ms), ROESY (rotating frame spectroscopy) (200 ms and 300 ms) and NOESY (Nuclear Overhuaser effect spectroscopy) (200 ms and 300 ms) experiments were acquired. The DQF-COSY^61^ was acquired with a data matrix size of 8 K × 512 and 1 s of recycle delay, 128 scans per *t*_1_ increment, and with the residual water signal attenuated by presaturation during the relaxation delay. TOCSY, ROESY and NOESY experiments were acquired with a data matrix size of 4 K × 512. The TOCSY was acquired with the MLEV17 spin-lock sequence^62^ and 1 s of relaxation time. Typically, 80 scans were acquired per *t*_1_ increment, and the residual water signal was removed by using the WATERGATE sequence^63^. NOESY and ROESY spectra^64,65^ were collected with typically 128 scans per *t*_1_ increment, with the residual water signal removed by the WATERGATE sequence and 1 s relaxation time.

Data were zero-filled, resolution-enhanced with phase-shifted sine bell (DQF-COSY) or square sine-bell window functions (TOCSY, NOESY and ROESY) optimized in each spectrum, baseline-corrected and processed with TopSpin 2.1. The proton resonances were assigned by standard sequential assignment processes^34^. The random-coil chemical shift values of H_α_ protons were obtained from tabulated data^34^; in the phosphorylated peptides, the values found in phosphorylated model random-coil peptides were used^66^. We used the observed down-field shifted tendency of the NH of phosphorylated residues to ensure that our peptides were also phosphorylated (Supplementary Tables S1–S13).

### Isothermal Titration Calorimetry (ITC)

Peptide binding to MDM2 was measured by using an Auto-iTC200 (Malvern Instruments, Malvern UK), as described^24,37^. Data acquisition was carried out with a reference power of 10 µcal/s, initial delay of 60 s, spacing between injections of 150 s. In a typical ITC experiment, the p53 peptide was loaded into a 40 μl syringe and the protein was placed into the sample cell. The sequence of injections consisted of one 0.5 μl injection followed by 19 injections of 2 μl each. Control experiments loading peptide into buffer solution were also carried out for each peptide. Experiments were performed with freshly prepared protein solutions, at 15 °C in the same buffer used in fluorescence experiments. ITC experiments were performed at 15 °C rather than 25 °C to avoid aggregation of MDM2.

Each peak was integrated using NITPIC software^67^. The areas of the peaks are related to the heat exchanged upon binding and/or dilution. This heat was corrected of the heat of dilution (i.e., the average heat of the injections after the equilibrium has reached the saturation). Titration with each peptide was repeated twice. In all peptides, the stoichiometry differed from the proposed 1:1-binding; this difference was probably due to the presence of inactive, and aggregated protein, as it has been observed in other binding studies of MDM2 with peptides^22,23^. We also tried to fit the data with Origin and PEAQ ITC software from Malvern and leaving the concentration of MDM2 as a floating parameter; we observed that: (i) the *K*_d_ and Δ*H* values are robust to these changes in cell concentration (the MDM2 protein); and (ii) using a 25% of the measured MDM2 concentration produced a binding stoichiometry close to 1:1 p53-peptide:MDM2. Therefore, we believe that only some proportion of fraction of loaded MDM2 was binding-competent but that the results obtained (*K*_d_ and Δ*H*) are a true reflection of the binding parameters.

### Kinetic measurements of p53-peptide binding to MDM2

The kinetics of the MDM2/p53-peptide interaction was measured at 15 °C using an Applied Photophysics stopped-flow fluorimeter in the same buffer as that used in the equilibrium fluorescence experiments. The experimental procedure has been described previously^23^. Association kinetics was measured under pseudo-first order conditions, by keeping the MDM2 concentration in excess of that of the peptide. The final peptide concentration in the mixing chamber was always 0.125 μM (from a 1:1 stock dilution in one of the stopped-flow syringes of 0.25 μM), and the final MDM2 concentration typically ranged from 1 μM to 3 μM (therefore, the protein concentration was always equal or larger than 8-times that of the peptide). Excitation wavelength was 280 nm with a cut-off filter of 315 nm on the emission side. The slit widths were 10 nm for excitation and emission.

At each MDM2 concentration a minimum of six traces were acquired; and the fitting in all cases for all peptides was carried out with the averaging of at least four traces. The averaged trace was always fitted to a single exponential function, which yielded, *k*_obs_. Fitting to a single exponential curve was carried out by using Kaleidagraph (Synergy Software); reported errors in *k*_obs_ are from the exponential fitting. The dissociation (*k*_off_) and association (*k*_on_) rate constants were obtained from the straight line:

$${k}_{obs}={k}_{on}\times [MDM2]+{k}_{off}.$$

Errors in both rates were from the linear fitting; the errors were always larger for *k*_off_ due to the extrapolation to the y-axis intercept.

### Replica exchange molecular dynamics simulations

Replica exchange molecular dynamics (REMD) simulations were carried out to explore the conformational landscapes of the isolated peptides, by using a methodology that we have described previously^49^. Briefly, all simulations were performed with the GROMACS 4.5^68,69^ MD suite. The starting structure for each simulation was an unfolded random conformation of the corresponding peptide, and was generated by running MD simulations at a higher temperature (350 K for 50 ns). We used the AMBER14SB force field^70^ with TIP3P water model^71^. The cut-off distances for the short-range neighbour list and van der Waals interactions were 1 nm and 1 nm, respectively. Prior to the production runs, each replica was equilibrated for 200 ps in the NVT and for 500 ps in the NPT ensembles. Coordinates between replicas were exchanged every 500 steps (10 ps) and the production run was carried out for 300 ns at 300 K in the NPT ensemble. The Verlet leapfrog algorithm was used to propagate the dynamics of the system at a time-step of 2 fs. Phosphorylation parameters were taken from previous studies^72^. The other details are similar to those used previously^49^.

For the characterization of conformational landscapes of peptides from our simulations we plotted 2D free energy surfaces (2D FESs) by using the following reaction coordinates: (i) Root Mean Square Deviation (RMSD) of the structure from the snapshot with the structure of the peptide in its bound form at the Cα atom level; and the Radius of gyration (*R*_g_) of that snapshot. The (RMSD, *R*_g_) values obtained for each snapshot were binned to get a histogram which was converted into FES by calculating (-ln(populations)). We refer to the basins on this plot as clusters (Fig. 2, Supplementary Fig. S1).

### Molecular dynamics simulations of MDM2-peptide complexes

The crystal structure of the N-terminal domain of MDM2 bound to the transactivation domain of p53 was obtained from the Protein Data Bank (PDB code 1YCR^16^). The p53 peptide was capped by acetyl and amide groups, while MDM2 was capped at its N- and C-termini by acetyl and N-methyl groups, respectively. Complexes of MDM2 with p53-pT18, p53-pS20, and p53-pT18pS20 peptides were generated by mutating the p53 peptide to the appropriate sequence. The mutations were performed by keeping the peptide backbone fixed and using the tleap module of AMBER 14^73^ to add the side chains of the mutated residues. Residue protonation states were determined by PDB2PQR^74^. The LEaP program in the AMBER 14 package was then used to solvate each system with TIP3P^71^ water molecules in a periodic truncated octahedron box, such that its walls were at least 10 Å away from the MDM2 complex, and for neutralization of charges with either sodium or chloride ions.

Three independent explicit-solvent MD simulations using different initial atomic velocities were carried out on each of the complexes of MDM2 with p53-WT, p53-pT18, p53-pS20, and p53-pT18pS20 peptides. Energy minimizations and MD simulations were carried out by the PMEMD module of AMBER 14 using the ff14SB force field, with a time step of 2 fs. Parameters for the phosphoresidues were used as described by Homeyer *et al*.^72^. All bonds involving hydrogen atoms were constrained by the SHAKE algorithm^75^. Non-bonded interactions were truncated at 9 Å while electrostatic interactions were treated by the particle mesh Ewald method^76^. Energy minimization was carried out for 500 steps using the steepest descent algorithm, followed by the conjugate gradient algorithm for another 500 steps. The system was then heated gradually to 300 K over 50 ps at constant volume before equilibration at a constant pressure of 1 atm for another 50 ps. Weak harmonic positional restraints with a force constant of 2.0 kcal mol^−1^ Å^−2^ were imposed on the non-hydrogen atoms of the solute during the minimization and these initial equilibration steps. Subsequent unrestrained equilibration (2 ns) and production (100 ns) runs were carried out at constant temperature and pressure. Temperature was maintained at 300 K using a Langevin thermostat^77^ with a collision frequency of 2 ps^−1^ while the pressure was maintained at 1 atm by a Berendsen barostat^78^ with a pressure relaxation time of 2 ps.

## Electronic supplementary material

Supplementary Information
