# Supplementary material for: Kinetic and thermodynamic effects of phosphorylation on p53 binding to MDM2
Source: Sci Rep. 2019 Jan 24;9:693. doi: 10.1038/s41598-018-36589-5 (PMC6345774; doi:10.1038/s41598-018-36589-5)

Kinetic and thermodynamic effects of phosphorylation on p53 binding to MDM2

Shilpa Yadahalli, José L. Neira, Christopher M. Johnson, Yaw Sing Tan, Pamela J. E. Rowling, Anasuya Chattopadhyay, Chandra S. Verma and Laura S. Itzhaki

SUPPLEMENTARY TABLES

The numbers in parenthesis after the chemical shifts of the Hα atoms correspond to the conformational shifts.

Table S1: Chemical shifts (δ, ppm from TSP) of p53 wild-type (pH 4.5, 283 K)

	NH	Hα	Hβ2	Hβ3	Hγ2	Hγ3	Hδ2	Hδ3	Hε	Hε	Hζ
Glu17	8.44	4.31 (0.02)	1.91		2.27						
Thr18	8.30	4.29 (-0.06)	4.15		1.13 (Me)						
Phe19	8.39	4.61 (-0.05)	3.05	3.15			7.20		7.30		
Ser20	8.20	4.31 (-0.19)	3.72	3.85							
Asp21	8.34	4.56 (-0.20)	2.63								
Leu22	8.02	4.11 (-0.27)	1.43		1.43		0.82 (Me)				
Trp23	7.90	4.54 (-0.16)	3.30				10.22 (NH); 7.26 (C2H); 7.47 (C7H); 7.57 (C4H); 7.18 (C5H); 7.28 (C6H)				
Lys24	7.61	4.04 (-0.32)	1.61		1.11				2.93		
Leu25	7.90	4.27 (-0.11)	1.61		1.61		0.91 (Me)				
Leu26	8.02	4.61 (0.23)	1.63		1.63		0.84 (Me)				
Pro27		4.38 (-0.06)	2.06		2.28		3.68	3.81			
Glu28	8.77	4.27 (-0.02)	2.02		2.34						
Asn29	8.41	4.68 (-0.07)	2.75								

Table S2: Chemical shifts (δ, ppm from TSP) of p53-pTpS (pH 4.5, 283 K)

	NH	Hα	Hβ2	Hβ3	Hγ2	Hγ3	Hδ2	Hδ3	Hε	Hε	Hζ
Glu17	8.42	4.32 (0.03)	1.90		2.34						
pThr18	8.40	4.37 (-0.06)	4.53		1.20 (Me)						
Phe19	8.39	4.70 (0.04)	2.97	3.20			7.22				
pSer20	8.60	4.44 (-0.16)	4.10	4.17							
Asp21	8.39	4.58 (-0.18)	2.68								
Leu22	8.04	4.10 (-0.28)	1.56		1.39		0.85 (Me)				
Trp23	7.90	4.56 (-0.14)	3.32				10.22 (NH); 7.30 (C2H); 7.47 (C7H); 7.57 (C4H); 7.18 (C5H); 7.28 (C6H)				
Lys24	7.55	4.05 (-0.31)	1.63		1.10				2.93		
Leu25	7.89	4.29 (-0.09)	1.63		1.63		0.90 (Me)				
Leu26	8.01	4.63 (0.25)	1.63		1.63		0.84 (Me)				
Pro27		4.35 (-0.09)	2.05		2.32		3.69	3.84			
Glu28	8.77	4.24 (-0.05)	2.03		2.37						
Asn29	8.41	4.70 (-0.05)	2.78								

Table S3: Chemical shifts (δ, ppm from TSP) of p53-pT (pH 4.5, 283 K)

	NH	Hα	Hβ2	Hβ3	Hγ2	Hγ3	Hδ2	Hδ3	Hε	Hε	Hζ
Glu17	8.42	4.29 (0.0)	1.92		2.30						
pThr18	8.44	4.33 (-0.10)	4.53		1.17 (Me)						
Phe19	8.40	4.69 (0.03)	2.99	3.20			7.21				
Ser20	8.31	4.27 (-0.23)	3.80	3.89							
Asp21	8.43	4.59 (-0.17)	2.64								
Leu22	8.01	4.10 (-0.28)	1.54		1.39		0.85 (Me)				
Trp23	7.88	4.52 (-0.18)	3.28				10.24 (NH); 7.29 (C2H); 7.47 (C7H); 7.57 (C4H); 7.22 (C5H); 7.29 (C6H)				
Lys24	7.59	4.05 (-0.31)	1.58		1.09				2.90		
Leu25	7.89	4.31 (-0.07)	1.62		1.62		0.90 (Me)				
Leu26	8.02	4.61 (0.23)	1.66		1.66		0.90 (Me)				
Pro27		4.40 (-0.04)	1.98	2.04	2.31		3.69	3.84			
Glu28	8.76	4.24 (-0.05)	2.01		2.35						
Asn29	8.41	4.67 (-0.08)	2.77								

Table S4: Chemical shifts (δ, ppm from TSP) of p53-pS (pH 4.5, 283 K)

	NH	Hα	Hβ2	Hβ3	Hγ2	Hγ3	Hδ2	Hδ3	Hε	Hε	Hζ
Glu17	8.43	4.35 (0.06)	1.90		2.31						
Thr18	8.27	4.34 (-0.01)	4.38		1.18 (Me)						
Phe19	8.57	4.53 (-0.13)	3.08				7.28		7.35		
pSer20	8.66	4.35 (-0.25)	4.13								
Asp21	8.15	4.51 (-0.25)	2.69								
Leu22	7.97	4.04 (-0.34)	1.61		1.37		0.84 (Me)				
Trp23	7.93	4.56 (-0.14)	3.25				10.16 (NH); 7.29 (C2H); 7.47 (C7H); 7.59 (C4H); 7.22 (C5H); 7.28 (C6H)				
Lys24	7.55	4.04 (-0.32)	1.65		1.17				2.90		
Leu25	7.83	4.29 (-0.09)	1.68		1.68		0.90 (Me)				
Leu26	7.93	4.56 (0.18)	1.61		1.61		0.90 (Me)				
Pro27		4.38 (-0.06)	2.05		2.32		3.67	3.84			
Glu28	8.78	4.23 (-0.06)	2.01		2.31						
Asn29	8.36	4.70 (-0.05)	2.74								

Table S5: Chemical shifts (δ, ppm from TSP) of p53-E18 (pH 4.5, 283 K)

	NH	Hα	Hβ2	Hβ3	Hγ2	Hγ3	Hδ2	Hδ3	Hε	Hε	Hζ
Glu17	8.42	4.21 (-0.08)	1.88		2.27						
Glu18	8.62	4.12 (-0.17)	1.81	1.99	2.16						
Phe19	8.21	4.61 (-0.05)	2.98	3.18			7.21		7.32		
Ser20	8.12	4.33 (-0.17)	3.75	3.86							
Asp21	8.44	4.55 (-0.21)	2.63								
Leu22	8.00	4.12 (-0.26)	1.55		1.55		0.90 (Me)				
Trp23	7.86	4.50 (-0.20)	3.33				10.23 (NH); 7.28 (C2H); 7.48 (C7H); 7.57 (C4H); 7.18 (C5H); 7.28 (C6H)				
Lys24	7.60	4.02 (-0.34)	1.59		1.10				2.91		
Leu25	7.87	4.26 (-0.12)	1.59		1.59		0.86 (Me)				
Leu26	8.00	4.63 (0.25)	1.63		1.63		0.92 (Me)				
Pro27		4.39 (-0.05)	1.98		2.30		3.66	3.83			
Glu28	8.76	4.21 (-0.08)	1.98		2.30						
Asn29	8.38	4.68 (-0.07)	2.74								

Table S6: Chemical shifts (δ, ppm from TSP) of p53-D20 (pH 4.5, 283 K)

	NH	Hα	Hβ2	Hβ3	Hγ2	Hγ3	Hδ2	Hδ3	Hε	Hε	Hζ
Glu17	8.41	4.29 (0.0)	1.90		2.27						
Thr18	8.25	4.33 (-0.02)	4.22		1.14 (Me)						
Phe19	8.42	4.57 (-0.09)	3.02	3.12			7.22		7.34		
Asp20	8.31	4.51 (-0.25)	2.67								
Asp21	8.23	4.55 (-0.21)	2.65								
Leu22	8.10	4.09 (-0.31)	1.55		1.34		0.83 (Me)				
Trp23	7.93	4.51 (-0.19)	3.28				10.21 (NH); 7.27 (C2H); 7.47 (C7H); 7.57 (C4H); 7.18 (C5H); 7.27(C6H)				
Lys24	7.57	4.02 (-0.34)	1.58		1.07				2.90		
Leu25	7.86	4.26 (-0.12)	1.57		1.57		0.87 (Me)				
Leu26	7.99	4.58 (0.20)	1.58		1.58		0.90 (Me)				
Pro27		4.37 (-0.07)	2.03		2.31		3.67	3.82			
Glu28	8.77	4.23 (-0.06)	2.00		2.33						
Asn29	8.39	4.70 (-0.05)	2.76								

Table S7: Chemical shifts (δ, ppm from TSP) of p3 p53-E18-D20 (pH 4.5, 283 K)

	NH	Hα	Hβ2	Hβ3	Hγ2	Hγ3	Hδ2	Hδ3	Hε	Hε	Hζ
Glu17	8.38	4.22 (-0.07)	1.86		2.37						
Glu18	8.55	4.22 (-0.07)	1.86		2.16						
Phe19	8.27	4.57 (-0.09)	2.98	3.18			7.22		7.34		
Asp20	8.29	4.55 (-0.21)	2.63								
Asp21	8.26	4.57 (-0.19)	2.71								
Leu22	8.09	4.10 (-0.28)	1.57		1.37		0.84 (Me)				
Trp23	7.91	4.51 (-0.19)	3.30				10.22 (NH); 7.29 (C2H); 7.49 (C7H); 7.57 (C4H); 7.18 (C5H); 7.28 (C6H)				
Lys24	7.57	4.00 (-0.36)	1.57		1.04				2.89		
Leu25	7.87	4.26 (-0.12)	1.60		1.63		0.88 (Me)				
Leu26	8.00	4.60 (0.22)	1.63		1.60		0.92 (Me)				
Pro27		4.38 (-0.06)	2.04		2.27		3.64	3.84			
Glu28	8.75	4.24 (-0.05)	1.98		2.34						
Asn29	8.40	4.69 (-0.06)	2.77								

Table S8: Chemical shifts (δ, ppm from TSP) of p53-E18-D20-E24-E25 (pH 4.5, 283 K)

	NH	Hα	Hβ2	Hβ3	Hγ2	Hγ3	Hδ2	Hδ3	Hε	Hε	Hζ
Glu17	8.37	4.23 (-0.06)	1.85		2.28						
Glu18	8.52	4.21 (-0.08)	1.83		2.20						
Phe19	8.32	4.56 (-0.10)	2.98	3.16			7.22		7.34		
Asp20	8.32	4.56 (-0.20)	2.67								
Asp21	8.31	4.55 (-0.21)	2.68								
Leu22	8.09	4.19 (-0.19)	1.46		1.46		0.80 (Me)				
Trp23	7.99	4.58 (-0.12)	3.31				10.18 (NH); 7.27 (C2H); 7.47 (C7H); 7.58 (C4H); 7.18 (C5H); 7.27 (C6H)				
Glu24	7.83	4.09 (-0.20)	1.77		2.05						
Glu25	8.09	4.19 (-0.10)	1.93		2.34						
Leu26	8.24	4.56 (0.18)	1.58		1.58		0.88 (Me)				
Pro27		4.41 (-0.03)	2.05		2.31		3.66	3.83			
Glu28	8.70	4.21 (-0.01)	1.97		2.34						
Asn29	8.44	4.68 (-0.07)	2.75								

Table S9: Chemical shifts (δ, ppm from TSP) of p53-E18-E20 (pH 4.5, 283 K)

	NH	Hα	Hβ2	Hβ3	Hγ2	Hγ3	Hδ2	Hδ3	Hε	Hε	Hζ
Glu17	8.43	4.19 (-0.10)	1.90		2.31						
Glu18	8.65	4.21 (-0.08)	1.84	2.07	2.19						
Phe19	8.26	4.60 (-0.06)	3.00	3.16			7.27		7.32		
Glu20	8.21	4.19 (-0.10)	1.94		2.29						
Asp21	8.34	4.51 (-0.25)	2.57								
Leu22	8.06	4.07 (-0.31)	1.52		1.38		0.83 (Me)				
Trp23	7.94	4.52 (-0.18)	3.32				10.22 (NH); 7.29 (C2H); 7.48 (C7H); 7.58 (C4H); 7.18 (C5H); 7.27(C6H)				
Lys24	7.60	4.05 (-0.31)	1.58		1.09				2.90		
Leu25	7.89	4.29 (-0.09)	1.60		1.60		0.83 (Me)				
Leu26	8.02	4.58 (0.20)	1.62		1.62		0.89 (Me)				
Pro27		4.37 (-0.07)	2.03		2.29		3.68	3.84			
Glu28	8.75	4.21 (-0.08)	2.00		2.31						
Asn29	8.41	4.70 (-0.05)	2.79								

Table S10: Chemical shifts (δ, ppm from TSP) of p53-D18 (pH 4.5, 283 K)

	NH	Hα	Hβ2	Hβ3	Hγ2	Hγ3	Hδ2	Hδ3	Hε	Hε	Hζ
Glu17	8.36	4.17 (-0.12)	1.86		2.25						
Asp18	8.46	4.58 (-0.18)	2.65								
Phe19	8.35	4.56 (-0.10)	3.00	3.18			7.22		7.32		
Ser20	8.28	4.23 (-0.27)	3.85								
Asp21	8.30	4.60 (-0.16)	2.63								
Leu22	7.97	4.09 (-0.29)	1.48		1.48		0.83 (Me)				
Trp23	7.90	4.56 (-0.14)	3.32				10.23 (NH); 7.29 (C2H); 7.49 (C7H); 7.58 (C4H); 7.18 (C5H); 7.28(C6H)				
Lys24	7.56	4.01 (-0.35)	1.63		1.06				2.90		
Leu25	7.89	4.29 (-0.09)	1.62		1.62		0.89 (Me)				
Leu26	8.00	4.60 (0.22)	1.62		1.62		0.93 (Me)				
Pro27		4.39 (-0.05)	1.98		2.32		3.66	3.84			
Glu28	8.75	4.25 (-0.04)	2.00		2.35						
Asn29	8.41	4.68 (-0.07)	2.77								

Table S11: Chemical shifts (δ, ppm from TSP) of p53-E20 (pH 4.5, 283 K)

	NH	Hα	Hβ2	Hβ3	Hγ2	Hγ3	Hδ2	Hδ3	Hε	Hε	Hζ
Glu17	8.45	4.28 (-0.01)	1.87		2.27						
Thr18	8.33	4.35 (0.0)	4.23		1.15 (Me)						
Phe19	8.48	4.48 (-0.18)	3.02				7.22		7.34		
Glu20	8.35	4.13 (-0.16)	1.92		2.22						
Asp21	8.20	4.46 (-0.30)	2.60								
Leu22	8.04	4.04 (-0.34)	1.49		1.35		0.82 (Me)				
Trp23	7.98	4.50 (-0.20)	3.31				10.20 (NH); 7.23 (C2H); 7.49 (C7H); 7.57 (C4H); 7.18 (C5H); 7.28(C6H)				
Lys24	7.59	4.07 (-0.29)	1.65		1.12				2.90		
Leu25	7.86	4.26 (-0.12)	1.61		1.61		0.86 (Me)				
Leu26	7.97	4.60 (0.22)	1.66		1.35		0.88 (Me)				
Pro27		4.36 (-0.08)	1.96		2.29		3.70	3.83			
Glu28	8.77	4.24 (-0.05)	1.96		2.32						
Asn29	8.39	4.66 (-0.09)	2.74								

Table S12: Chemical shifts (δ, ppm from TSP) of p53-D18-E20 (pH 4.5, 283 K)

	NH	Hα	Hβ2	Hβ3	Hγ2	Hγ3	Hδ2	Hδ3	Hε	Hε	Hζ
Glu17	8.39	4.25 (-0.04)	1.79		2.26						
Asp18	8.52	4.68 (-0.08)	2.67								
Phe19	8.28	4.54 (-0.12)	3.04	3.10			7.25		7.37		
Glu20	8.31	4.15 (-0.14)	1.93		2.26						
Asp21	8.16	4.62 (-0.14)	2.55								
Leu22	8.01	4.13 (-0.25)	1.48		1.33		0.76 (Me)				
Trp23	7.96	4.54 (-0.16)	3.29				10.22 (NH); 7.28 (C2H); 7.48 (C7H); 7.58 (C4H); 7.18 (C5H); 7.27(C6H)				
Lys24	7.56	4.09 (-0.27)	1.58		1.08				2.90		
Leu25	7.87	4.37 (-0.01)	1.58		1.58		0.82 (Me)				
Leu26	7.99	4.66 (0.28)	1.56		1.56		0.86 (Me)				
Pro27		4.37 (-0.07)	2.04		2.30		3.68	3.84			
Glu28	8.75	4.23 (-0.06)	1.97		2.34						
Asn29	8.41	4.74 (-0.01)	2.75								

Table S13: Chemical shifts (δ, ppm from TSP) of p53-D18-D20 (pH 4.5, 283 K)

	NH	Hα	Hβ2	Hβ3	Hγ2	Hγ3	Hδ2	Hδ3	Hε	Hε	Hζ
Glu17	8.38	4.22 (-0.07)	1.84		2.26						
Asp18	8.50	4.61 (-0.15)	2.71								
Phe19	8.30	4.53 (-0.13)	3.05	3.14			7.22		7.30		
Asp20	8.36	4.55 (-0.21)	2.71								
Asp21	8.17	4.55 (-0.21)	2.67								
Leu22	8.09	4.10 (-0.28)	1.55		1.37		0.81 (Me)				
Trp23	7.95	4.53 (-0.17)	3.31				10.22 (NH); 7.27 (C2H); 7.47 (C7H); 7.58 (C4H); 7.18 (C5H); 7.27(C6H)				
Lys24	7.53	4.09 (-0.27)	1.58		1.06				2.93		
Leu25	7.89	4.26 (-0.12)	1.61		1.60		0.91 (Me)				
Leu26	8.01	4.61 (0.23)	1.66		1.60		0.89 (Me)				
Pro27		4.35 (-0.09)	2.02		2.30		3.67	3.84			
Glu28	8.76	4.26 (-0.03)	2.03		2.38						
Asn29	8.42	4.68 (-0.07)	2.75								

SUPPLEMENTARY FIGURES

FIGURE 1: **TFE titrations monitored by CD**: (A) The far-UV CD spectra of selected peptides in aqueous solution (50 mM phosphate buffer, pH 6.8) at 5 º C. (B) Main panel: TFE titration for p53-E18-D20 at 5 ºC (50 mM phosphate buffer, pH 6.8). The line is the fit of the data to a two-state equation[32,33]. Inset: Far-UV CD spectra of p53-E18-D20 at different TFE concentrations; the arrow indicates the increasing concentration of TFE.

FIGURE 2: **Far-UV CD spectrum of p53-E18-D20-E24-E25**: The far-UV CD spectra of the quadruple mutant in aqueous solution (50 mM phosphate buffer, pH 6.8) at 5 º C.

FIGURE 3: **NMR structural characterization of selected p53 peptides**: NOEs are classified into strong, medium or weak according to the height of the bar underneath the sequence; signal intensity was judged by visual inspection from the ROESY experiments with 200 ms of mixing time. The corresponding H_α NOEs with the following H_δ of a proline residue are indicated by an open bar in the row corresponding to the $\alpha N(i, i+1)$ contacts. The dotted lines indicate NOE contacts which could not be unambiguously assigned due to signal overlap or diagonal proximity of the chemical shifts. The numbering of residues corresponds to that of the whole sequence of p53.

FIGURE 4: **Chemical shifts of the peptides calculated from the REMD simulations**: Experimental values are shown in red (from the Tables above), and those calculated from simulation are in blue. We used the program SPARTA[36] to calculate chemical shifts from the simulations. The values shown are the mean values for each residue from 3000 structures collected from 300 ns of simulation data.

FIGURE 5: **Ramachandran plots (x-axis: ϕ, y-axis: ψ) of phosphorylated residues**: The plots show how the dihedral angle distribution of residues in phosphorylated residues changes with their conformation in p53-WT. The colour bars indicate the populations, with blue indicating highest density and yellow lowest density of structures.

FIGURE 6: **Conformational landscapes of the peptides containing the phosphomimics**: 2D FES surfaces of p53-WT and peptides containing phosphomimics sampled during the REMD simulations. The X-axis shows the RMSD of each conformation of a peptide calculated from the bound form of the WT peptide from the crystal structure 1YCR[16]. The Y-axis shows the radius of gyration (R_g) of the peptide. Representative structures from significantly populated clusters are shown in a cartoon format. Key binding residues Phe19, Trp23 and Leu26 are shown as sticks. The colours represent the populations of the peptide conformations and the color scheme used in shown on the right side; blue and yellow correspond to the highest and lowest density of conformations sampled in our REMD simulations.

FIGURE 7: **Equilibrium fluorescence binding titrations**: Binding monitored by fluorescence (the difference in fluorescence intensity of p53 peptide in complex relative to uncomplexed peptide) for (A) p53-PSpT and (B) p53-pS. The line through the experimental data is the fit of the data to equation (1). Experiments were carried out at 15 °C.

FIGURE 8: **Fluorescence spectra of the equilibrium binding titrations**: Fluorescence spectra (without subtraction of the spectrum of the corresponding isolated peptide) of the titration for p53-pT. Experiments were carried out at 15 °C.

Fig. S1 (Yadahalli et al)

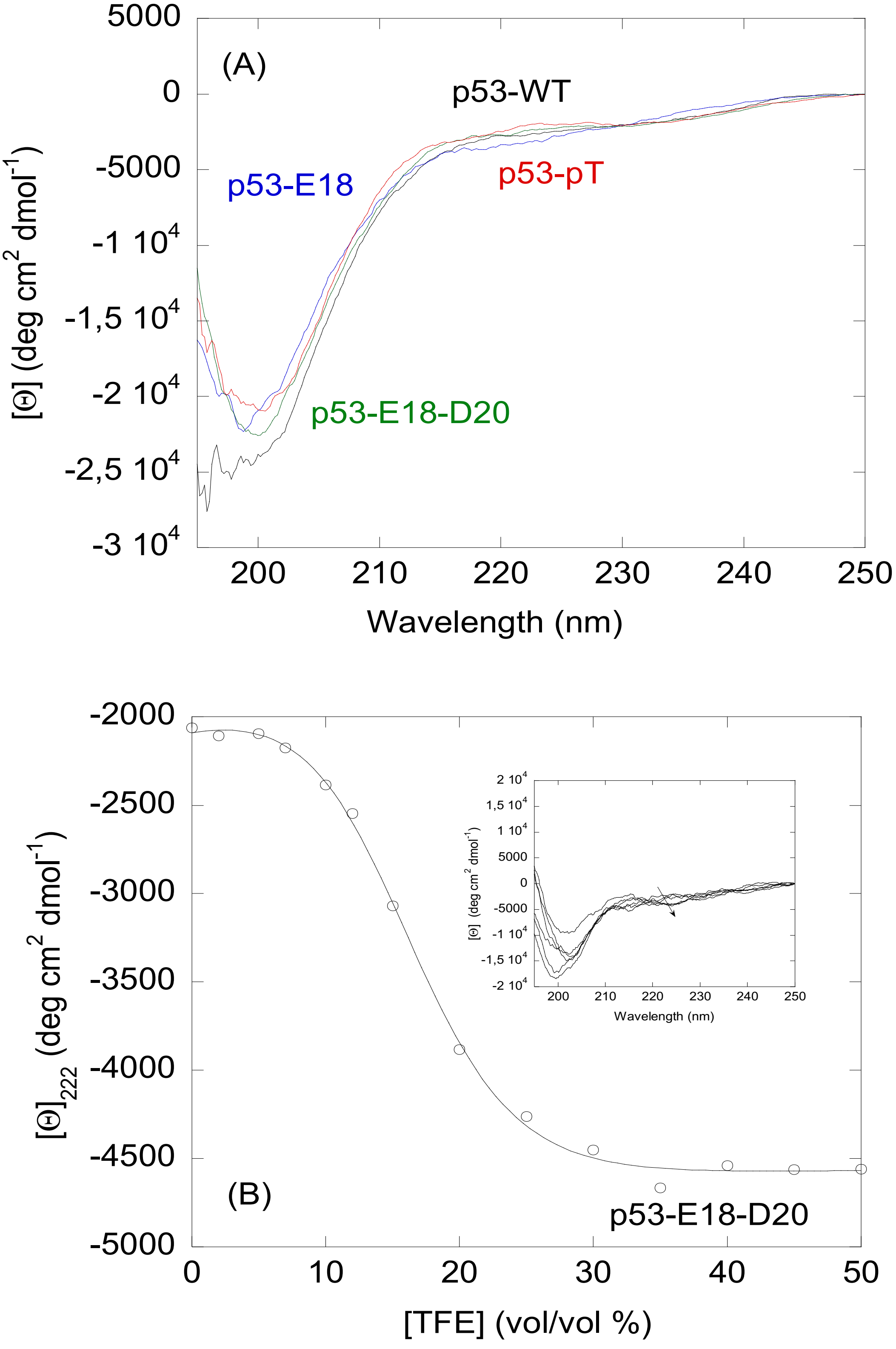

Fig. S2 (Yahadalli et al)

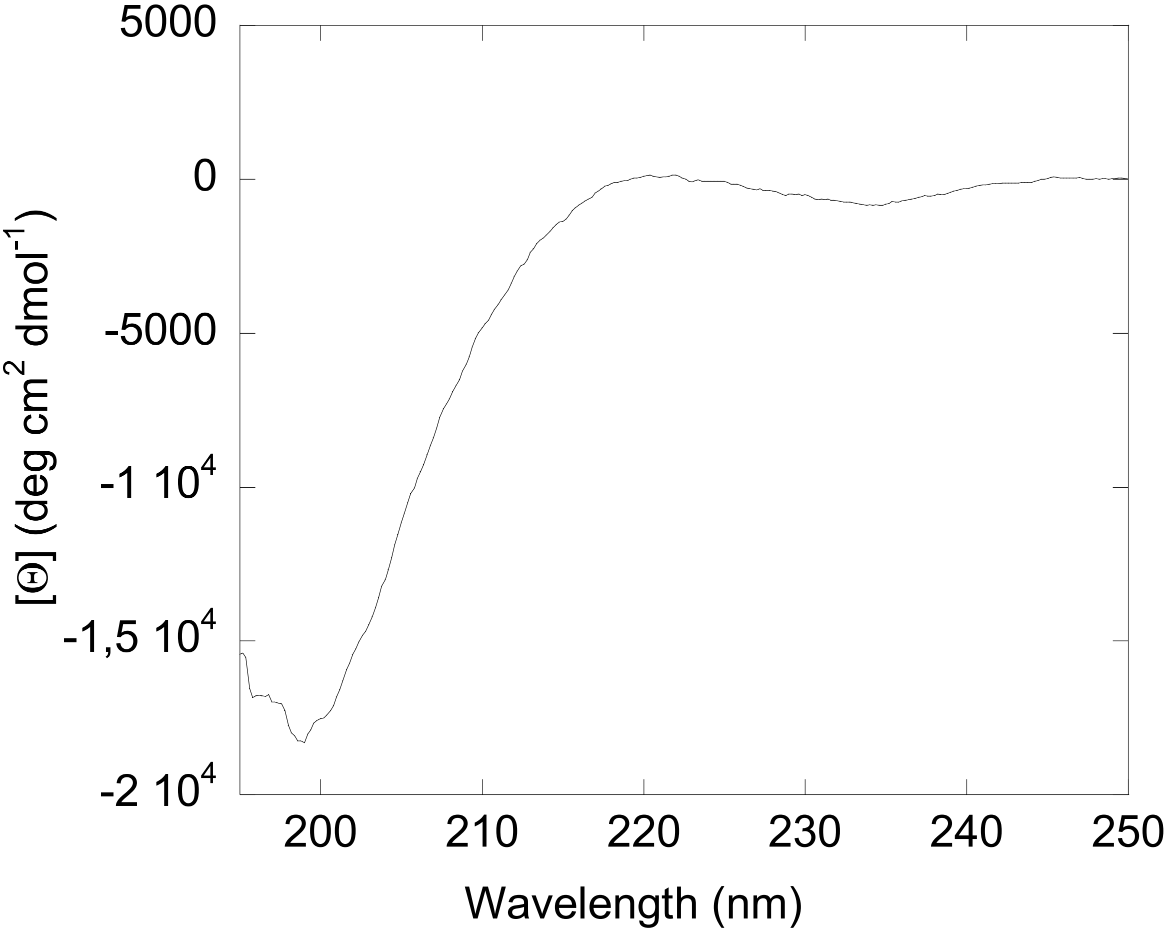

Fig. S3 (Yadahalli et al)

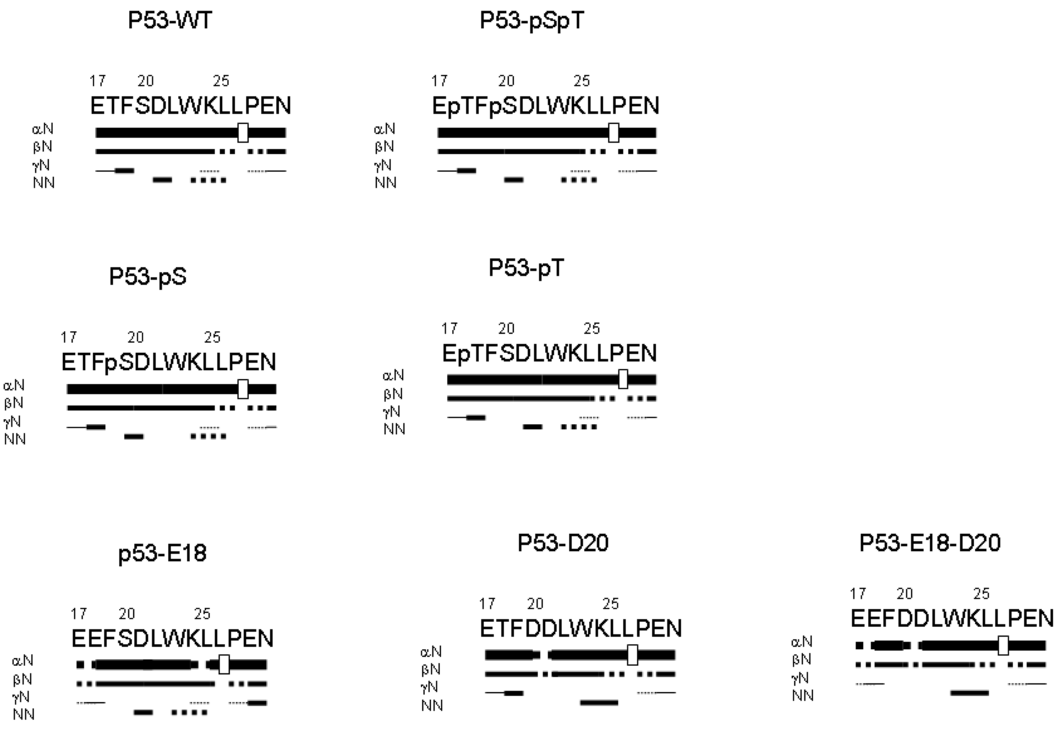

Fig. S4 (Yadahalli et al.)

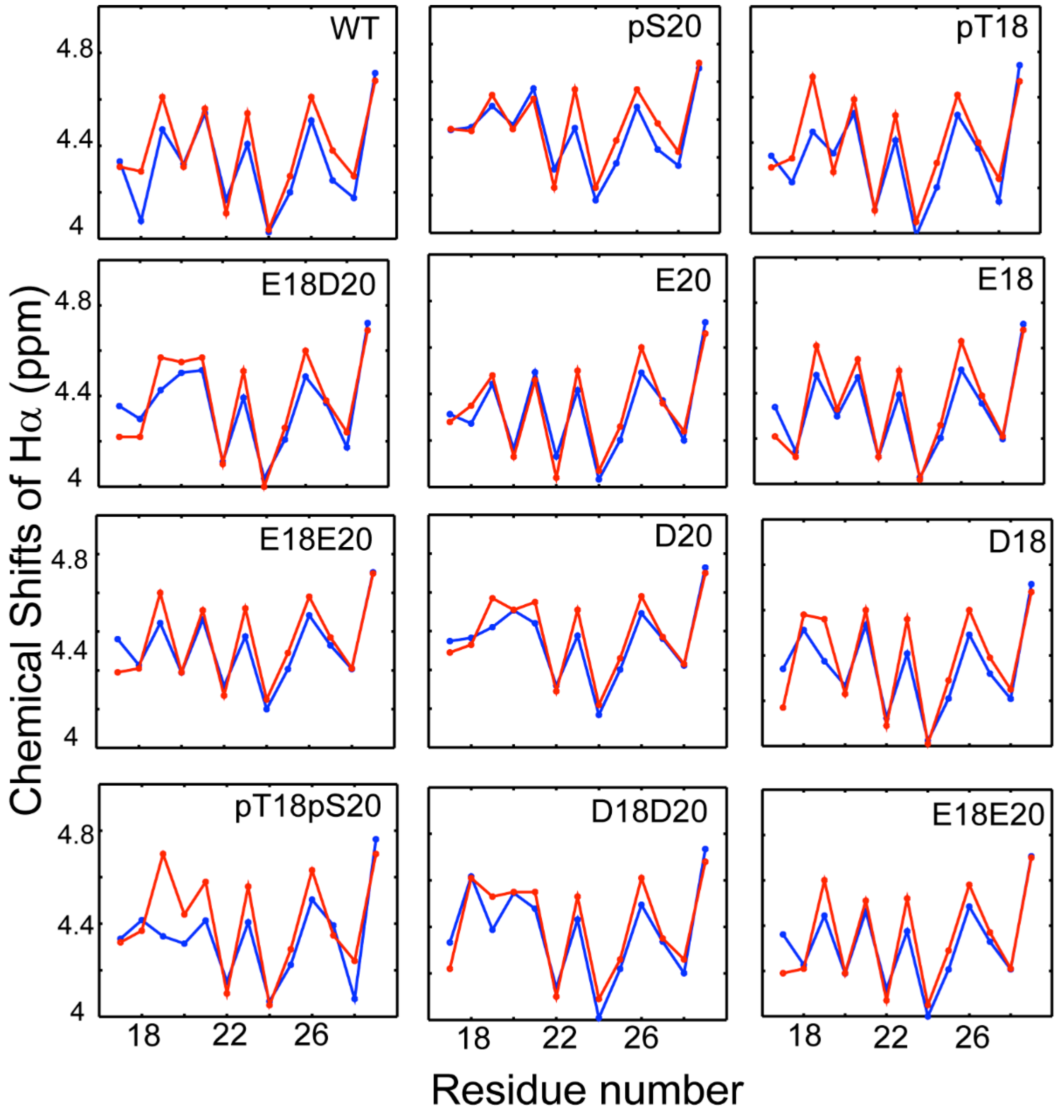

Fig. S5 (Yadahalli et al.)

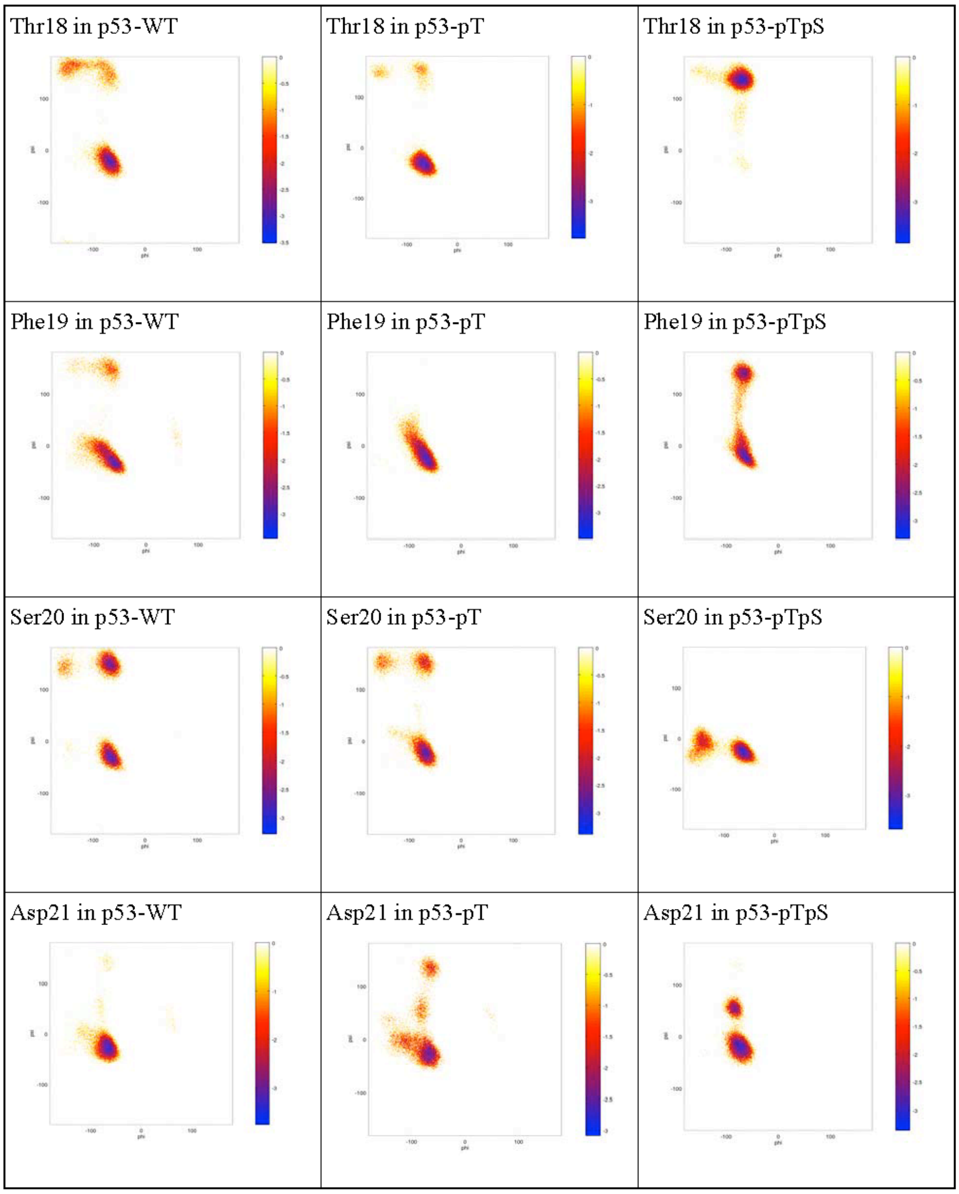

Fig. S6 (Yadahalli et al.)

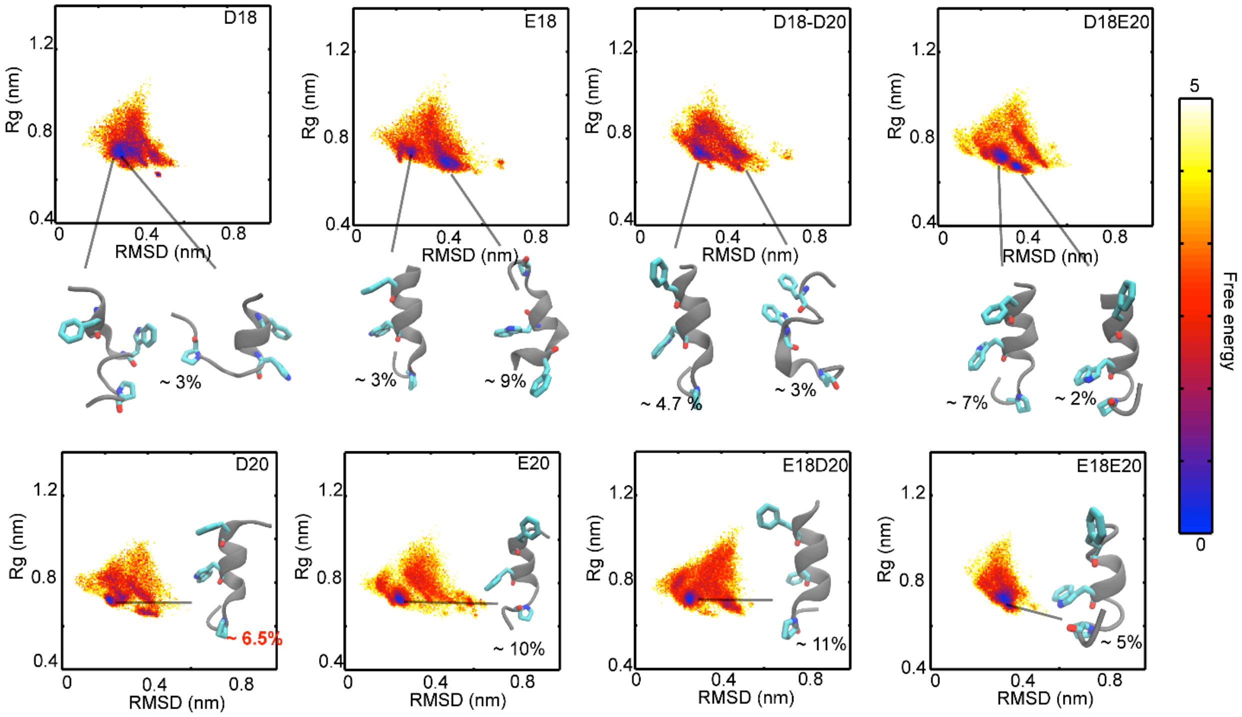

Fig. S7 (Yadahalli et al)

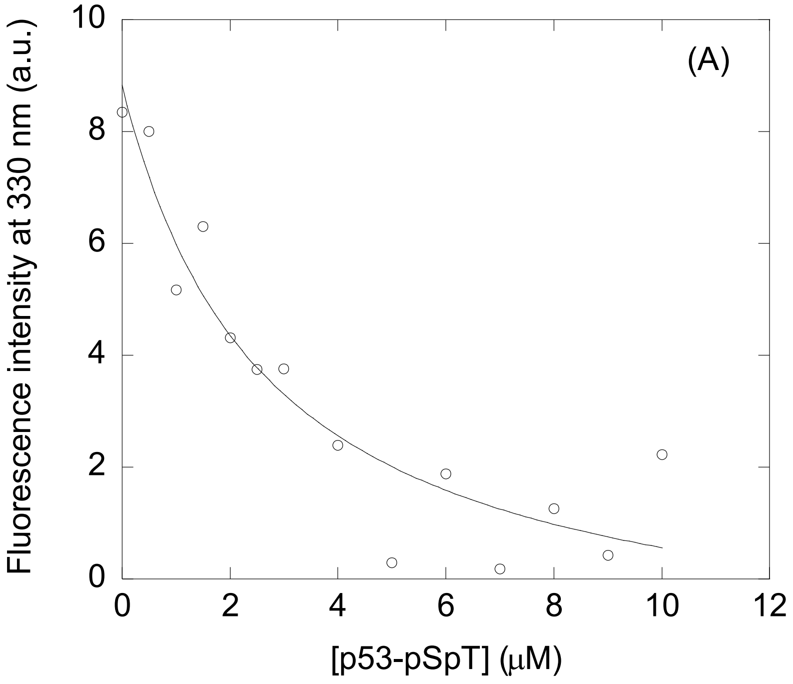

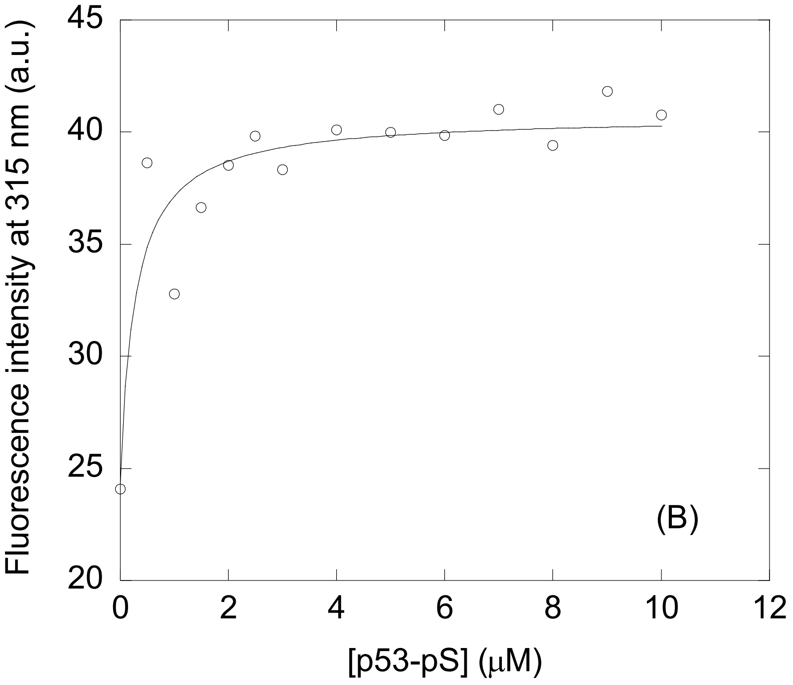

Fig. S8 (Yadahalli et al)

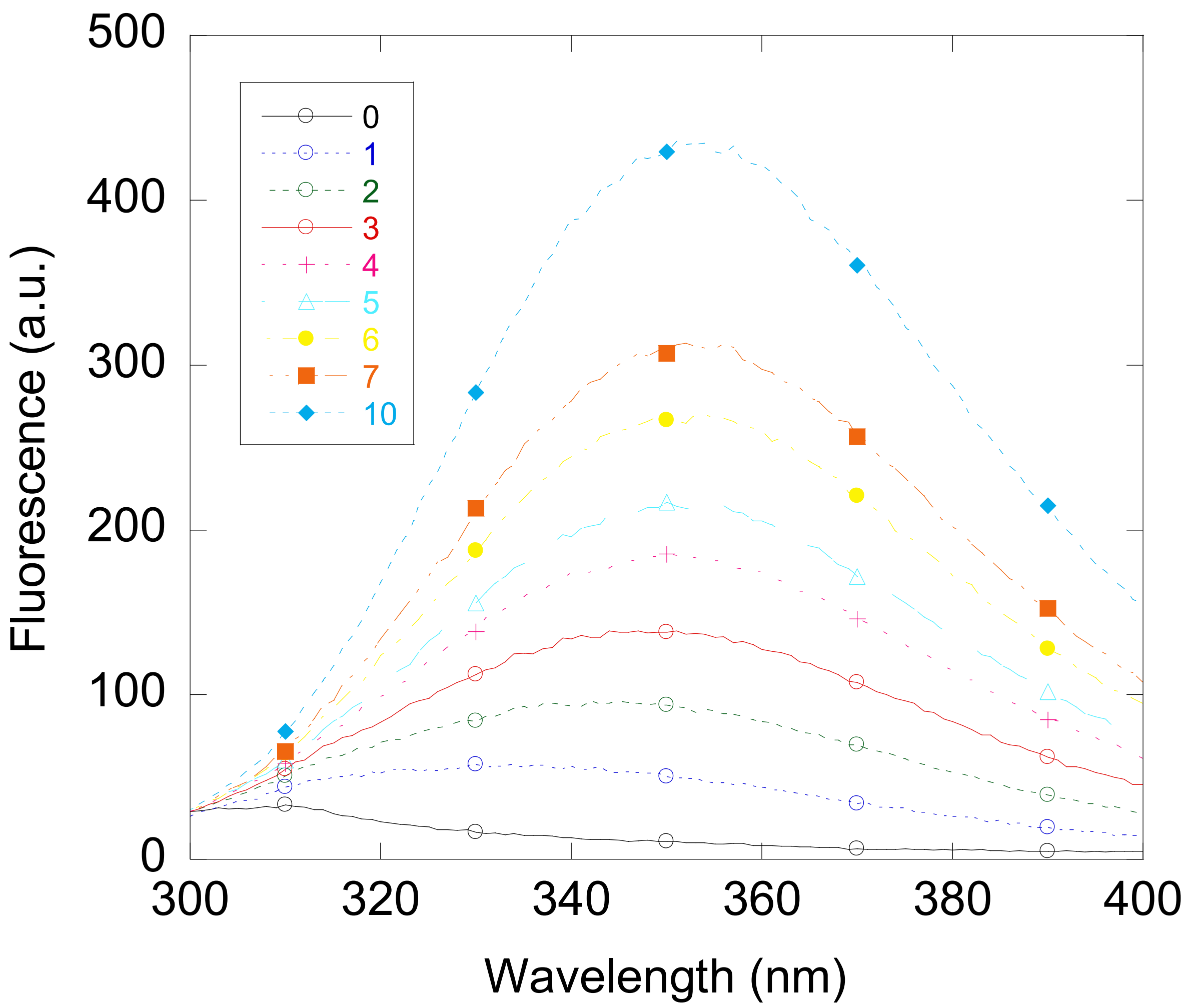

Supplement: Supplementary file 1 — Supplementary Information [file 41598_2018_36589_MOESM1_ESM.pdf]
